# Supplementary material for: Optineurin provides a mitophagy contact site for TBK1 activation
Source: EMBO J. 2024 Jan 29;43(5):754–79. doi: 10.1038/s44318-024-00036-1 (PMC10907724; doi:10.1038/s44318-024-00036-1)
Supplement: Supplementary file 1 — Appendix [file 44318_2024_36_MOESM1_ESM.pdf]

Appendix

Optineurin provides a mitophagy contact site for TBK1 activation.

Koji Yamano, Momoha Sawada, Reika Kikuchi, Kafu Nagataki, Waka Kojima, Ryu Endo, Hiroki Kinefuchi, Atsushi Sugihara, Tomoshige Fujino, Aiko Watanabe, Keiji Tanaka, Gosuke Hayashi, Hiroshi Murakami, Noriyuki Matsuda

Table of Contents		page
Appendix Table S1	Reagents and cell lines	2,3
Appendix Table S2	Antibodies	4,5
Appendix Table S3	Plasmids and siRNAs	6-8
Appendix Fig S1	Ubiquitination of autophagy adaptors upon Parkin-mediated mitophagy	9
Appendix Fig S2	Time course of TBK1 autophosphorylation upon Parkin-mediated mitophagy	10
Appendix Fig S3	Kinetic parameters of the OPTN monobodies	11

Appendix Table S1. Reagents and cell lines

REAGENT or RESOURCE	SOURCE	IDENTIFIER
Reagents		
Antimycin A	Sigma-Aldrich	Cat. # A8674
Oligomycin	Calbiochem	Cat. # 495455-10MGCN
Valinomycin	Sigma-Aldrich	Cat. # V0627
Bafilomycin A1	Calbiochem	Cat. # 196000
DMEM	Sigma-Aldrich	Cat. # D5796
McCoy's 5A	Gibco	Cat. # 16600-082
FBS	biowest	Cat. # S1820
MEM Non-Essential Amino Acids Solution	Gibco	Cat. # 11140-050
Sodium Pyruvate	Gibco	Cat. # 11360-070
Penicilin-Streptomycin-Glutamine	Gibco	Cat. # 10378-016
GlutaMAX Supplement	Gibco	Cat. # 35050-061
Trypsin-EDTA (0.25%)	Gibco	Cat. # 25200-056
FuGENE6	Promega	Cat. # E269A
Lipofectamine LTX	Invitrogen	Cat. # 15338100
Lipofectamine RNAiMAX	Invitrogen	Cat. # 13778150
CHAPS	Sigma-Aldrich	Cat. # C3023
cOmplete, EDTA-free Protease Inhibitor Cocktail	Roche	Cat. # 11873580001
PhosSTOP, Phosphatase Inhibitor Cocktail	Roche	Cat. # 04906845001
BX-795 (TBK1 inhibitor)	abcam	Cat. # ab142016
4% PFA Solution	Wako	Cat. # 163-20145
Gelatin	Sigma-Aldrich	Cat. # G9391
Triton X-100	MP Biomedicals	Cat. # 807426
Tween 20	nacalai tesque	Cat. # 35624-15
NuPAGE 4-12% Bis-Tris gel	Invitrogen	Cat. # NP0323BOX
Difco Skim Milk	BD Biosciences	Cat. # 232100
Phusion High-Fidelity DNA polymerase	Thermo Fisher Scientific	Cat. # F-530L
DNA Ligation Kit	TaKaRa	Cat. # 6023
Western Lightning Plus-ECL	PerkinElmer	Cat. # NEL105001EA
ECL Prime Western Blotting Detection Reagents	GE Healthcare	Cat. # RPN2232
BL21-CodonPlus(DE3)-RIL Competent Cells	Agilent Technology	Cat. # 230245

FuGENE® HD Transfection Reagent	Promega	Cat. # E2311
TCEP (Tris(2-carboxyethyl)phosphine hydrochloride	Sigma	Cat. # C4706-10G
Ni-NTA Agarose	QIAGEN	Cat. # 30230
Glutathione Sepharose 4B	cytiva	Cat. # 17075605
PD MidiTrap™ G-25	cytiva	Cat. # 28918008
PreScission™ Protease	cytiva	Cat. # 27084301
DTT (1,4-dithiothreitol)	Roche	Cat. # 10708984001
Imidazole	FUJIFILM	Cat. # 095-00015
L-Glutathione reduced	Sigma	Cat. # G4251-25G
GFP-Trap Agarose	ChromoTek GmbH	Cat. # gta-20
Epoxomicin	PEPTIDE INSTITUTE, INC.	Cat. # 4381-v
Z-Leu-Leu-Leu-H (aldehyde) MG-132	PEPTIDE INSTITUTE, INC.	Cat. # 3175-v
Concanamycin A	Adipogen Life Sciences	Cat. # BVT-0237-C100
Recombinant human His6 USP2 catalytic domain	BostonBiochem	Cat. # E-506
Cell lines		
WT HeLa	ATCC	CCL-2,2
HeLa AAVS-Parkin	This study	
FIP200 KO HeLa	(Vargas et al., 2019)	
ATG5 KO HeLa	(Nezich et al., 2015)	
ATG9A KO HeLa	(Nezich et al., 2015)	
Penta (OPTN/NDP52/TAX1BP1/p62/NBR1) KO HeLa	(Lazarou et al., 2015)	
TBK1 KO HeLa	This study	
FIP200/TBK1 DKO HeLa	This study	
ATG9A/TBK1 DKO HeLa	This study	
HEK293T	ATCC	CRL-3216
HCT116	(Yamano et al., 2018)	
TBK1-/- HCT116	(Yamano et al., 2020)	

Appendix Table S2. Antibodies

REAGENT or RESOURCE	SOURCE	IDENTIFIER
Antibodies for immunobloting		
Rabbit monoclonal anti-NAK/TBK1 (EP611Y)	abcam	Cat. # ab40676, RRID:AB_776632
Mouse monoclonal anti-β-Actin (6D1)	MBL	Cat. # M177-3, RRID:AB_10697039
Rabbit polyclonal anti-TOMM20	Proteintech	Cat. # 11802-1-AP, RRID:AB_2207530
Rabbit monoclonal anti-NDP52 (D1E4A)	Cell Signaling	Cat. # 60732, RRID:AB_2732810
Rabbit monoclonal anti-APG5L/ATG5 (EPR1755(2))	abcam	Cat. # ab108327, RRID:AB_2650499
Mouse monoclonal anti-DDDDK (FLA-1)	MBL	Cat. # M185-3L, RRID:AB_11123930
Rabbit polyclonal anti-LC3B	Sigma-Aldrich	Cat. # L7543, RRID:AB_796155
Rabbit polyclonal anti-SQSTM1/p62	MBL	Cat. # PM045, RRID:AB_1279301
Rabbit monoclonal anti-Phospho-SQSTM1/p62 (S403) (D8D6T)	Cell Signaling	Cat. # 39786, RRID:AB_2799162
Mouse monoclonal anti-NBR1 (4BR)	Santa Cruz	Cat. # sc-130380, RRID:AB_2149402
Rabbit monoclonal anti-TAX1BP1 (D1D5)	Cell Signaling	Cat. # 5105S, RRID:AB_11178939
Mouse monoclonal anti-Multi Ubiquitin (FK2)	MBL	Cat. # D058-3, RRID:AB_592937
Rabbit polyclonal anti-AZI2/NAP1	Proteintech	Cat. # 15042-1-AP, RRID:AB_2878103
Rabbit monoclonal anti-SINTBAD/TBKBP1 (D1A5)	Cell Signaling	Cat. # 8605S, RRID:AB_10839270
Rabbit polyclonal anti-GFP	abcam	Cat. # ab6556, RRID:AB_305564
Rabbit monoclonal anti-Phospho TBK1 (S172) (D52C2)	Cell Signaling	Cat. # 5483, RRID:AB_10693472
Rabbit monoclonal anti-ATG9A (EPR2450(2))	abcam	Cat. # ab108338, RRID:AB_10863880
Rabbit monoclonal anti-FIP200 (D10D11)	Cell Signaling	Cat. # 12436S, RRID:AB_2797913
Rabbit polyclonal anti-OPTN	Proteintech	Cat. # 10837-1-AP, RRID:AB_2156665
Rabbit monoclonal anti-Phospho-OPTN (S177)	Cell Signaling	Cat. # 57548S, RRID:AB_2799529
Anti-Mouse IgG horseradish peroxidase-linked	Promega	Cat. # W402B, RRID:AB_430834
Anti-Rabbit IgG horseradish peroxidase-linked	Promega	Cat. # W401B, RRID:AB_430833
Antibodies for immunostaining		
Mouse monoclonal anti-TOMM20 (F-10)	Santa Cruz	Cat. # sc-17764, RRID:AB_628381
Rabbit polyclonal anti-OPTN	Proteintech	Cat. # 10837-1-AP, RRID:AB_2156665
Rabbit polyclonal anti-NDP52	abcam	Cat. # ab68588, RRID:AB_RRID:AB_1640255
Mouse monoclonal anti-DDDDK (FLA-1)	MBL	Cat. # M185-3L, RRID:AB_11123930

Rabbit polyclonal anti-TOMM20	Proteintech	Cat. # 11802-1-AP, RRID:AB_2207530
Guinea pig polyclonal anti-p62	MBL	Cat. # PM066, RRID:AB_10952738
Rabbit polyclonal anti-SQSTM1/p62	MBL	Cat. # PM045, RRID:AB_1279301
Mouse monoclonal anti-Multi Ubiquitin (FK2)	MBL	Cat. # D058-3, RRID:AB_592937
Rabbit monoclonal anti-Phospho TBK1 (S172) (D52C2)	Cell Signaling	Cat. # 5483, RRID:AB_10693472
Rabbit monoclonal anti-TAX1BP1 (D1D5)	Cell Signaling	Cat. # 5105S, RRID:AB_11178939
Mouse monoclonal anti-NBR1 (4BR)	Santa Cruz	Cat. # sc-130380, RRID:AB_2149402
Rabbit polyclonal anti-Ferritin	Rockland	Cat. # 200-401-090-0100, RRID:AB_2612112
Rabbit monoclonal anti-Phospho-OPTN (S177)	Cell Signaling	Cat. # 57548S, RRID:AB_2799529
Rat monoclonal anti-HA (3F10)	Roche	Cat. # 11867423001, RRID:AB_390918
Rabbit polyclonal anti-NBR1	Gift from Komatsu	
Rabbit polyclonal anti-WIPI2	Sigma-Aldrich	Cat. # SAB4200400
Goat polyclonal anti-HSP60 (N-20)	Santa Cruz	Cat. # sc-1052, RRID:AB_631683
Goat anti-Rabbit IgG Alexa Fluor 488 conjugated	Invitrogen	Cat. # A-11034, RRID:AB_2576217
Goat anti-Rabbit IgG Alexa Fluor 568 conjugated	Invitrogen	Cat. # A-11036, RRID:AB_10563566
Goat anti-Rabbit IgG Alexa Fluor 647 conjugated	Invitrogen	Cat. # A-21245, RRID:AB_2535813
Goat anti-Mouse IgG Alexa Fluor 488 conjugated	Invitrogen	Cat. # A-11029, RRID:AB_2534088
Goat anti-Mouse IgG Alexa Fluor 568 conjugated	Invitrogen	Cat. # A-11031, RRID:AB_144696
Goat anti-Mouse IgG Alexa Fluor 647 conjugated	Invitrogen	Cat. # A-21236, RRID:AB_2535805
Goat anti-Rat IgG Alexa Fluor 647 conjugated	Invitrogen	Cat. # A-21247, RRID:AB_141778
Goat anti-Guinea pig IgG Alexa Fluor 647 conjugated	Invitrogen	Cat. # A-21450, RRID:AB_141882
Donkey anti-Goat igG Alexa Fluor 647 conjugated	Thermo Fisher Scientific	Cat. # A-21447, RRID:AB_2535864

Appendix Table S3. Plasmids and siRNAs

REAGENT or RESOURCE	SOURCE
Plasmid DNA	
pcDNA3.1(+)	Invitrogen (V79020)
pMXs-puro_YFP	Yamano et al., 2020
pMXs-puro_3FLAG-OPTN	Yamano et al., 2020
pBABE-puro_3FLAG-OPTN	This study
pMXs-puro_3FLAG-NDP52	Yamano et al., 2020
pBABE-puro_3FLAG-NDP52	This study
pMXs-puro_3FLAG-OPTN(4LA)	Yamano et al., 2020
pMXs-puro_3FLAG-OPTN(F178A)	Yamano et al., 2020
pMXs-puro_3FLAG-OPTN(4LA, F178A)	Yamano et al., 2020
pBMNz/mEGFP-Optineurin	Lazarou et al. 2015
pMXs-puro_pSu9-mCherry-3FLAG	This study
pMXs-puro_3HA-WIPI1	This study
pBMNz-YFP-Parkin	Lazarou et al., 2015
pRetroQ-mt-Keima	Gift from Chunxin Wang
pBABE-puro_Untag-Parkin	Yamano et al., 2020
pZDonor-AAVS1-Puro-EF1p-untag-Parkin	This study
phAG_OPTN(S473E)	Yamano et al., 2020
phAG_OPTN(M44Q/L54Q, S473E)	Yamano et al., 2020
pHA-Ash_6Ub	Yamano et al., 2020
pUMVC (Gag-Pol)	Gift from Chunxin Wang
pCMV-VSV-G (VSV-G)	Gift from Chunxin Wang
pMXs-puro_EYFP-P2A-EcoRI	This study
pMXs-puro_EYFP-P2A-hOpt_TBK1 WT	This study
pMXs-puro_EYFP-P2A-hOpt_TBK1 R47H	This study
pMXs-puro_EYFP-P2A-hOpt_TBK1 D135N	This study
pMXs-puro_EYFP-P2A-hOpt_TBK1 S172A	This study
pMXs-puro_EYFP-P2A-hOpt_TBK1 G217R	This study
pMXs-puro_EYFP-P2A-hOpt_TBK1 R357Q	This study
pMXs-puro_EYFP-P2A-hOpt_TBK1 M559R	This study
pMXs-puroYFP-TEV-OPTN monobody clone #1	This study

pMXs-puroYFP-TEV-OPTN monobody clone #2	This study
pMXs-puroYFP-TEV-OPTN monobody clone #3	This study
pMXs-puroYFP-TEV-OPTN monobody clone #4	This study
pMXs-puroYFP-TEV-OPTN monobody clone #5	This study
pMXs-puroYFP-TEV-OPTN monobody clone #6	This study
pMXs-puroYFP-TEV-OPTN monobody clone #7	This study
pMXs-puroYFP-TEV-OPTN monobody clone #8	This study
pMXs-puroYFP-TEV-OPTN monobody clone #9	This study
pMXs-puroYFP-TEV-OPTN monobody clone #10	This study
pMXs-puroYFP-TEV-OPTN monobody clone #11	This study
pMXs-puroYFP-TEV-OPTN monobody clone #12	This study
pET21a(+)_OPTN monobody clone #2	This study
pET21a(+)_OPTN monobody clone #3	This study
pET21a(+)_OPTN monobody clone #4	This study
pET21a(+)_OPTN monobody clone #5	This study
pET21a(+)_OPTN monobody clone #6	This study
pET21a(+)_OPTN monobody clone #7	This study
pET21a(+)_OPTN monobody clone #8	This study
pET21a(+)_OPTN monobody clone #9	This study
pET16b_OPTN monobody clone #2	This study
pET16b_OPTN monobody clone #3	This study
pET16b_OPTN monobody clone #4	This study
pET16b_OPTN monobody clone #5	This study
pET16b_OPTN monobody clone #6	This study
pET16b_OPTN monobody clone #7	This study
pET16b_OPTN monobody clone #8	This study
pET16b_OPTN monobody clone #9	This study
pGEX6P1_Opt_TBK1(677-729aa)	This study
pET16b_His10-NdeI-EGFP	This study
pET16b_His10-Opt_OPTN(26-196aa, E50K)-EGFP	This study
pMXs-puro_EGFP-TEV-OPTN Monobody #3	This study
pMXs-puro_EGFP-TEV-OPTN Monobody #4	This study
pGEX6P1_Opt_OPTN(26-119aa)-sortase-His6	This study
pGEX6P1_Opt_OPTN(26-196aa)-sortase-His6	This study

pGEX6P1_Opt_OPTN(133-196aa)-sortase-His6	This study
Oligonucleotides	
siRNA FIP200 #94 (GCCUAGAACAACUAACGAAtt)	Thermo Fisher Scientific
siRNA FIP200 #95 (GUCGUCUCCUAAUCCUAUAtt)	Thermo Fisher Scientific
siRNA ATG9A #05 (CCACAAACGUGAGCUGACAtt)	Thermo Fisher Scientific
siRNA ATG9A #06 (GAAUAUGCAUCCACAGAGAtt)	Thermo Fisher Scientific
siRNA OPTN #s19719 (GGAGACUGUUGGAAGCGAAtt)	Thermo Fisher Scientific
siRNA NDP52 #s19996 (CCUUCAUGUGGGUUACUUUtt)	Thermo Fisher Scientific
siRNA TAX1BP1 #s16986 (CUGAUACACUGGAACACGAtt)	Thermo Fisher Scientific
siRNA p62 #8878 (GAUCUGCGAUGGCUGCAAU)	Dharmacon
siRNA NBR1 #4077 (GGAGUGGAUUUACCAGUUA)	Dharmacon

Appendix Figure S1

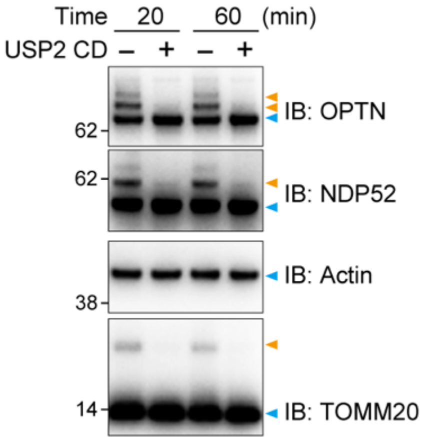

Appendix Figure S1. Ubiquitination of autophagy adaptors upon Parkin-mediated mitophagy

Parkin-expressing HeLa cells were treated with valinomycin in the presence of bafilomycin for 1 hr. The cells solubilized with 2% CHAPS were incubated with or without recombinant USP2 catalytic domain (USP2-CD) for 20 or 60 min and analyzed by immunoblotting. The light blue and orange arrowheads indicate unmodified and ubiquitinated protein bands, respectively.

Appendix Figure S2

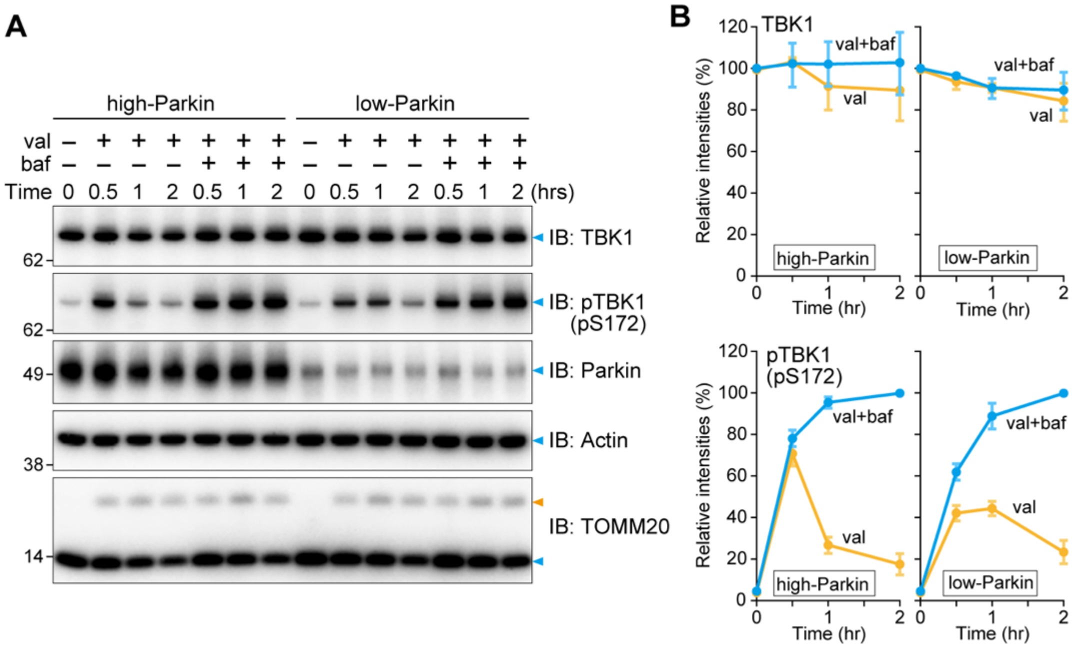

Appendix Figure S2. Time course of TBK1 autophosphorylation upon Parkin-mediated mitophagy

A High-Parkin denotes HeLa cells with the *PARKIN* gene integrated in the AAVS-locus under the CMV promoter, whereas low-Parkin denotes HeLa cells stably expressing Parkin following retrovirus infection (See Materials and Methods for details). Both groups of cells were treated with valinomycin (val) and bafilomycin (baf) for the indicated times. Total cell lysates were analyzed by immunoblotting (IB). The light blue and orange arrowheads indicate unmodified and ubiquitinated protein bands, respectively.

B The levels of proteins indicated in (A) were quantified. TBK levels without val treatment were set to 100. pTBK1(pS172) levels after 2 hrs of val and baf treatment were set to 100. Error bars represent mean ± s.d. of three independent experiments.

Appendix Figure S3

A

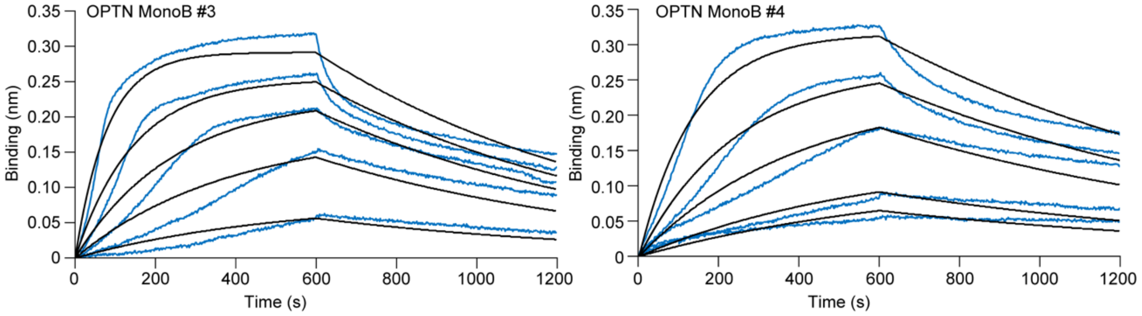

B

Names	K_D (nM)	k_{on} (1/Ms) $\times 10^4$	k_{off} (1/s) $\times 10^{-4}$
OPTN MonoB #3	4.56	27.81	12.69
OPTN MonoB #4	6.19	15.84	9.81

Appendix Figure S3. Kinetic parameters of the OPTN monobodies

A Kinetic parameters for the OPTN monobodies were determined by Bio-layer interferometry. A biotin-labeled OPTN (26-196 aa) was immobilized on a sensor chip and various concentrations (40, 20, 10, 5, and 2.2 nM) of monobodies (MonoB) #3 and #4 were used for the kinetic analyses. Experimental data are shown in blue and the 1:1 binding model is shown in black.

B Kinetic parameters of the OPTN monobodies.

References

Lazarou M, Sliter DA, Kane LA, Sarraf SA, Wang C, Burman JL, Sideris DP, Fogel AI, Youle RJ (2015) The ubiquitin kinase PINK1 recruits autophagy receptors to induce mitophagy. *Nature* 524: 309-314

Nezich CL, Wang C, Fogel AI, Youle RJ (2015) MiT/TFE transcription factors are activated during mitophagy downstream of Parkin and Atg5. *The Journal of cell biology* 210: 435-450

Vargas JNS, Wang C, Bunker E, Hao L, Maric D, Schiavo G, Randow F, Youle RJ (2019) Spatiotemporal Control of ULK1 Activation by NDP52 and TBK1 during Selective Autophagy. *Molecular cell* 74: 347-362 e346

Yamano K, Wang C, Sarraf SA, Munch C, Kikuchi R, Noda NN, Hizukuri Y, Kanemaki MT, Harper W, Tanaka K *et al* (2018) Endosomal Rab cycles regulate Parkin-mediated mitophagy. *eLife* 7: e31326

Yamano K, Kikuchi R, Kojima W, Hayashida R, Koyano F, Kawawaki J, Shoda T, Demizu M, Tanaka K et al (2020) Critical role of mitochondrial ubiquitination and the OPTN-ATG9A axis in mitophagy. *The Journal of cell biology* 219: e201912144
