# Supplementary figures and images for: Optineurin provides a mitophagy contact site for TBK1 activation
Source: EMBO J. 2024 Jan 29;43(5):754–79. doi: 10.1038/s44318-024-00036-1 (PMC10907724; doi:10.1038/s44318-024-00036-1)

Fig 1A

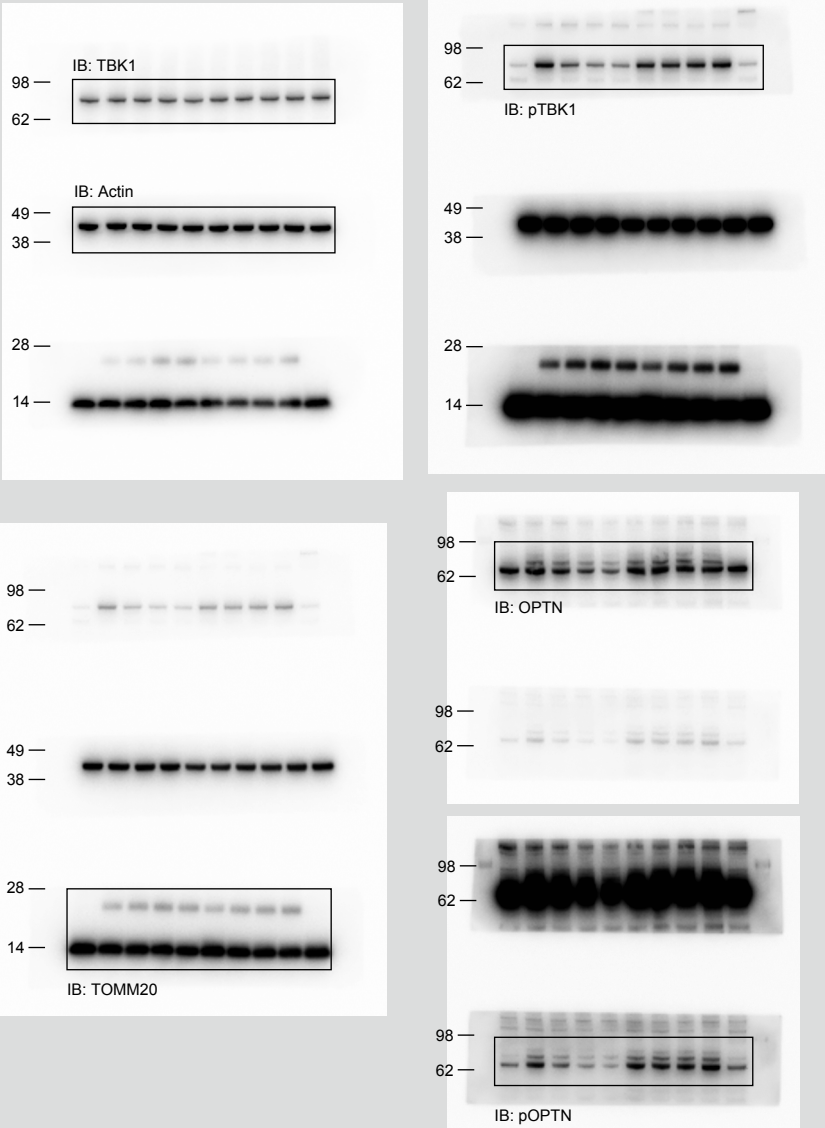

Supplement: Supplementary file 2 — Source Data Fig. 1 [file 44318_2024_36_MOESM2_ESM.zip › Figure1/1A/SourceData_Fig1A.pdf]

Fig 1G

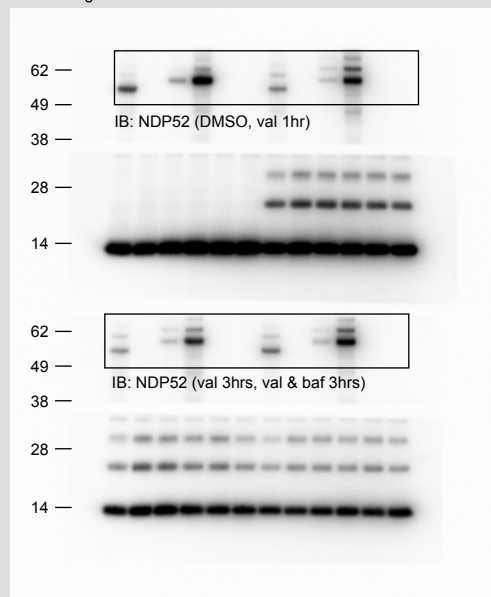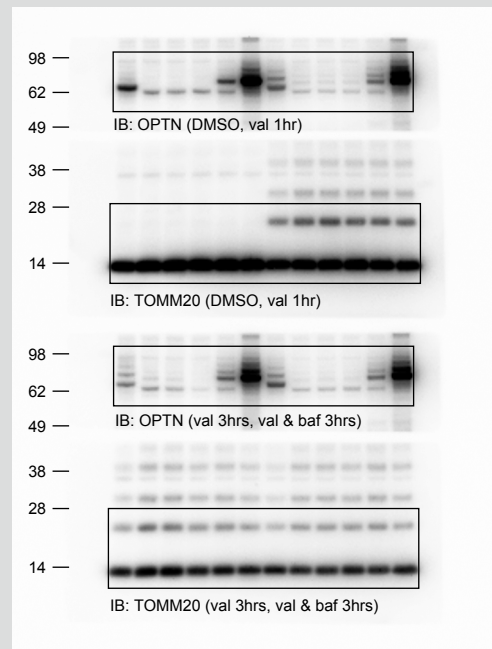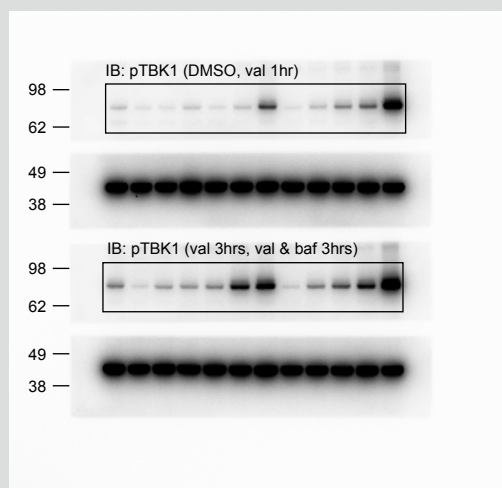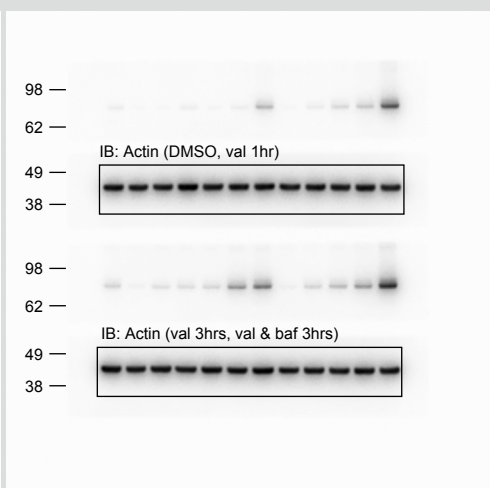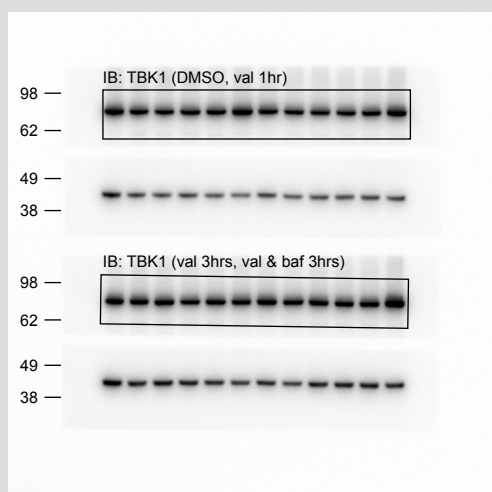

Supplement: Supplementary file 2 — Source Data Fig. 1 [file 44318_2024_36_MOESM2_ESM.zip › Figure1/1G/SourceData_Fig1G.pdf]

Fig 11

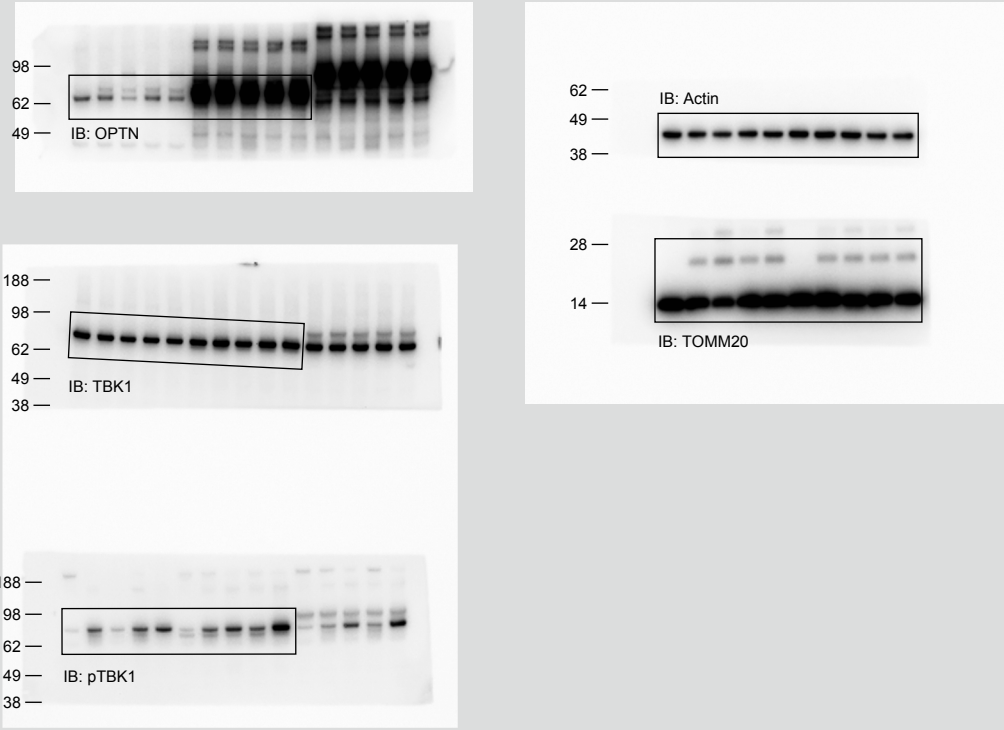

Supplement: Supplementary file 2 — Source Data Fig. 1 [file 44318_2024_36_MOESM2_ESM.zip › Figure1/1I/SourceData_Fig1I.pdf]

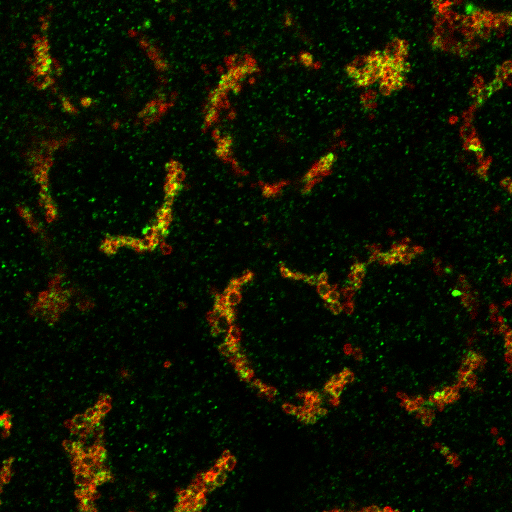

Supplement: Supplementary file 2 — Source Data Fig. 1 [file 44318_2024_36_MOESM2_ESM.zip › Figure1/1L/SourceData_Fig1L_homo.tif]

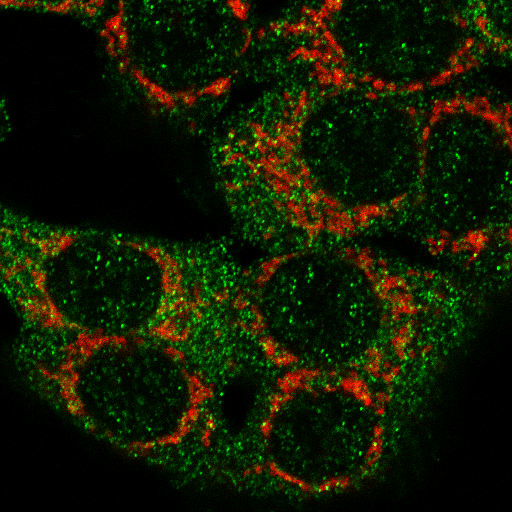

Supplement: Supplementary file 2 — Source Data Fig. 1 [file 44318_2024_36_MOESM2_ESM.zip › Figure1/1L/SourceData_Fig1L_cytosol.tif]

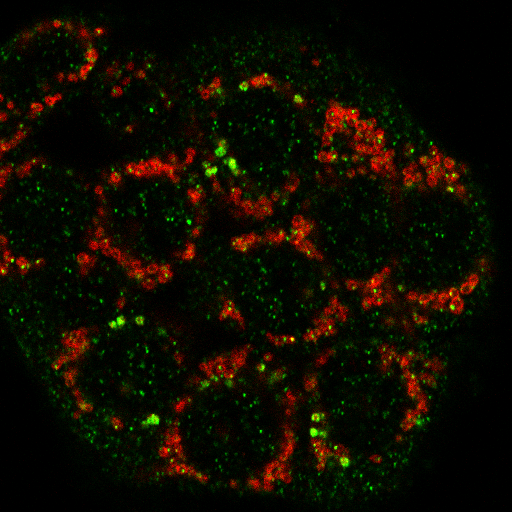

Supplement: Supplementary file 2 — Source Data Fig. 1 [file 44318_2024_36_MOESM2_ESM.zip › Figure1/1L/SourceData_Fig1L_hetero.tif]

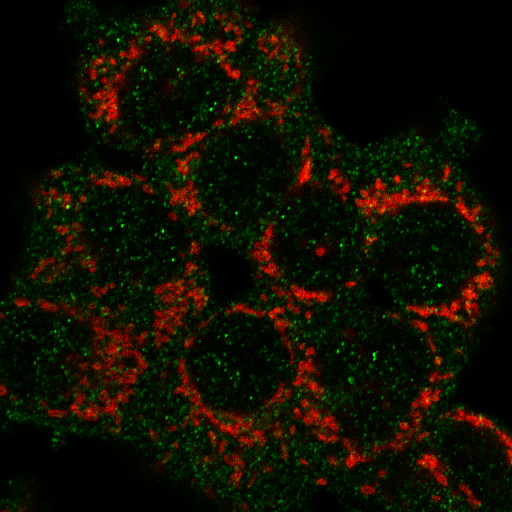

Supplement: Supplementary file 2 — Source Data Fig. 1 [file 44318_2024_36_MOESM2_ESM.zip › Figure1/1K/SourceData_Fig1K_TBK1KO_DMSO_OPTN.tif]

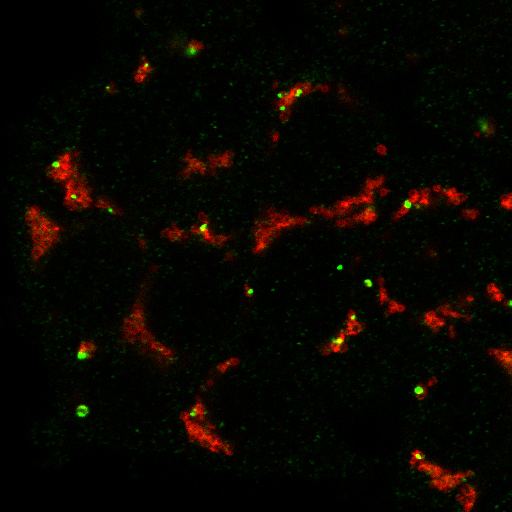

Supplement: Supplementary file 2 — Source Data Fig. 1 [file 44318_2024_36_MOESM2_ESM.zip › Figure1/1K/SourceData_Fig1K_WT_val1hr_NDP52.tif]

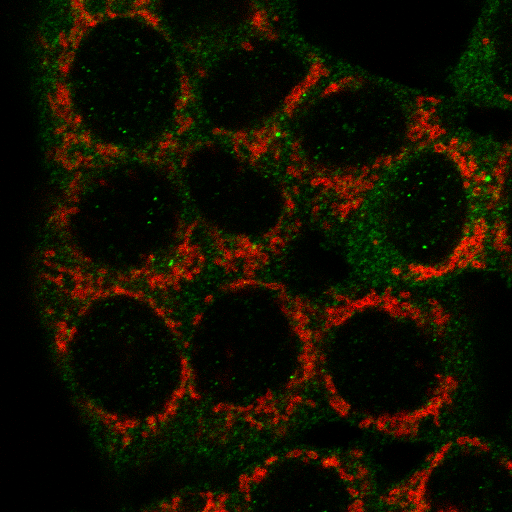

Supplement: Supplementary file 2 — Source Data Fig. 1 [file 44318_2024_36_MOESM2_ESM.zip › Figure1/1K/SourceData_Fig1K_TBK1KO_DMSO_NDP52.tif]

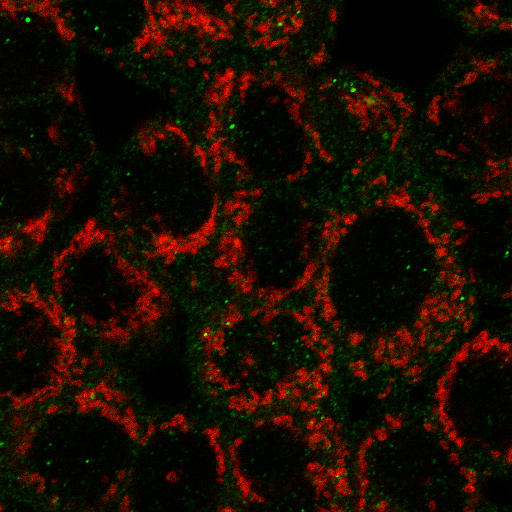

Supplement: Supplementary file 2 — Source Data Fig. 1 [file 44318_2024_36_MOESM2_ESM.zip › Figure1/1K/SourceData_Fig1K_WT_DMSO_NDP52.tif]

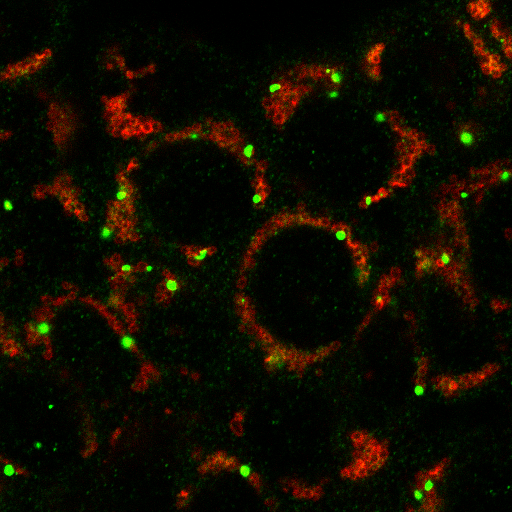

Supplement: Supplementary file 2 — Source Data Fig. 1 [file 44318_2024_36_MOESM2_ESM.zip › Figure1/1K/SourceData_Fig1K_TBK1KO_val1hr_NDP52.tif]

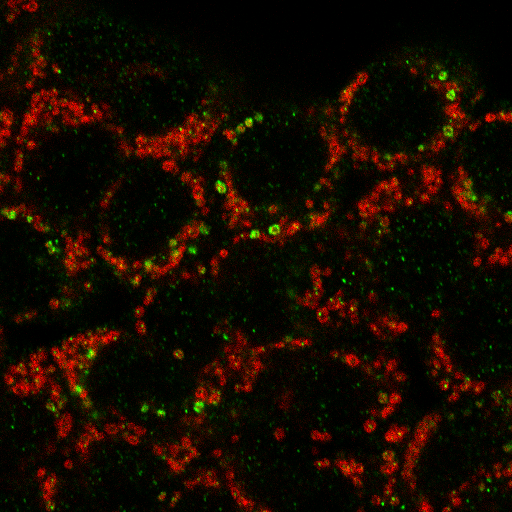

Supplement: Supplementary file 2 — Source Data Fig. 1 [file 44318_2024_36_MOESM2_ESM.zip › Figure1/1K/SourceData_Fig1K_WT_val1hr_OPTN.tif]

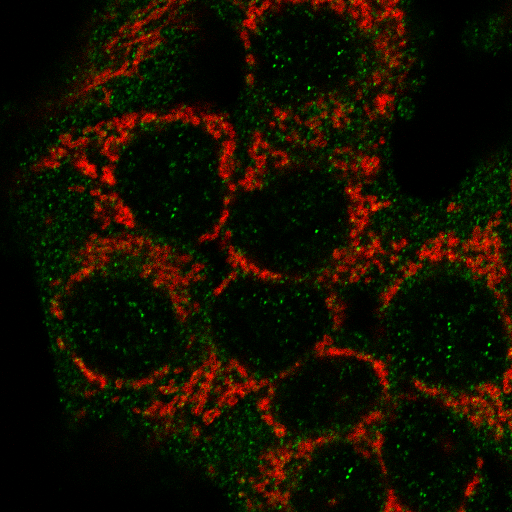

Supplement: Supplementary file 2 — Source Data Fig. 1 [file 44318_2024_36_MOESM2_ESM.zip › Figure1/1K/SourceData_Fig1K_WT_DMSO_OPTN.tif]

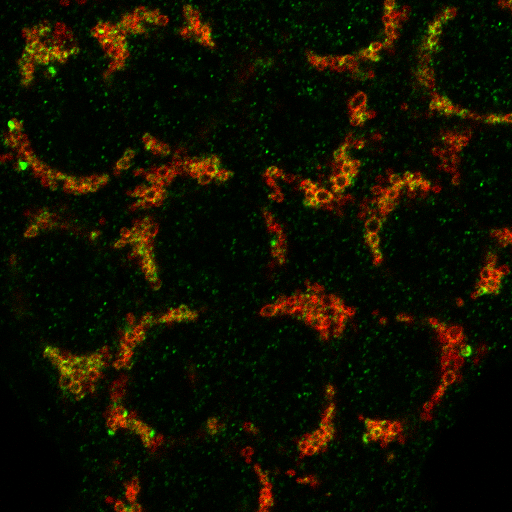

Supplement: Supplementary file 2 — Source Data Fig. 1 [file 44318_2024_36_MOESM2_ESM.zip › Figure1/1K/SourceData_Fig1K_TBK1KO_val1hr_OPTN.tif]

Fig 1E

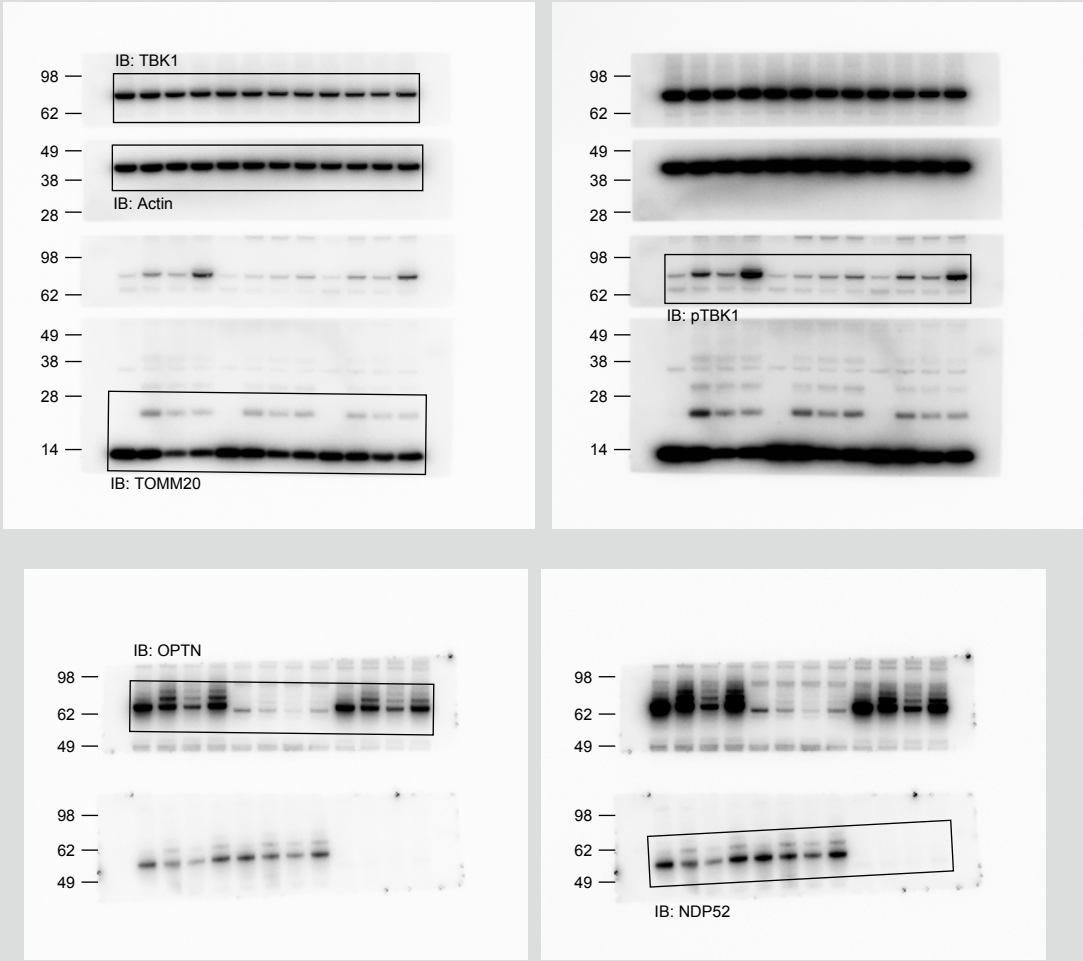

Supplement: Supplementary file 2 — Source Data Fig. 1 [file 44318_2024_36_MOESM2_ESM.zip › Figure1/1E/SourceData_Fig1E.pdf]

Fig 1C

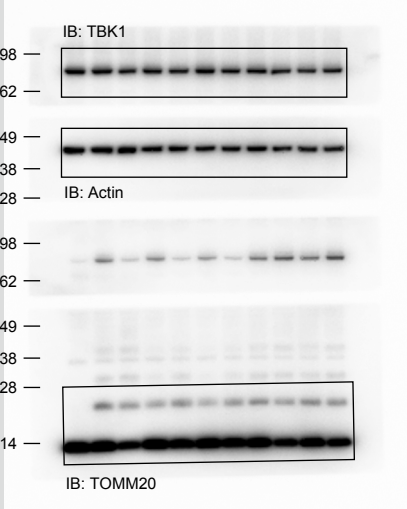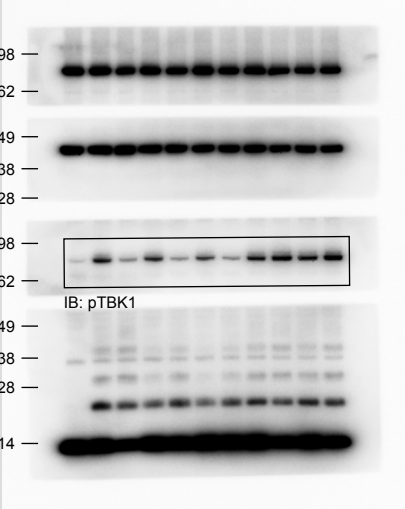

Supplement: Supplementary file 2 — Source Data Fig. 1 [file 44318_2024_36_MOESM2_ESM.zip › Figure1/1C/SourceData_Fig1C.pdf]

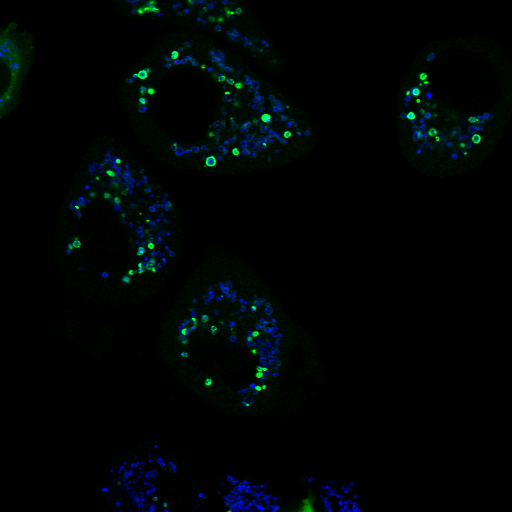

Supplement: Supplementary file 3 — Source Data Fig. 2 [file 44318_2024_36_MOESM3_ESM.zip › Figure2/2G/SourceData_Fig2G_hetero.tif]

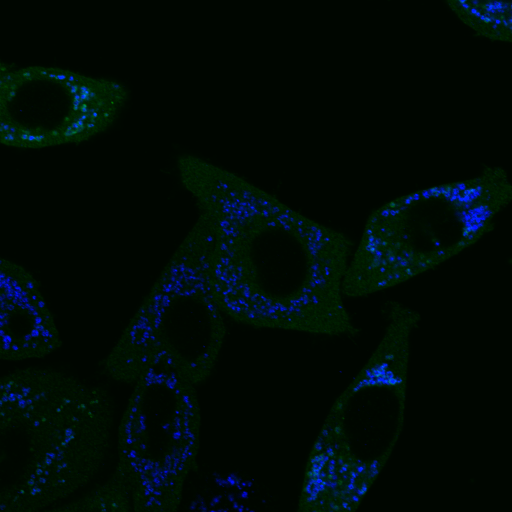

Supplement: Supplementary file 3 — Source Data Fig. 2 [file 44318_2024_36_MOESM3_ESM.zip › Figure2/2G/SourceData_Fig2G_cytosol.tif]

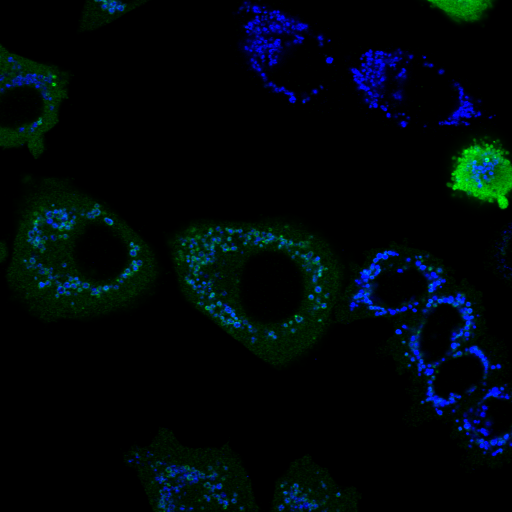

Supplement: Supplementary file 3 — Source Data Fig. 2 [file 44318_2024_36_MOESM3_ESM.zip › Figure2/2G/SourceData_Fig2G_homo.tif]

Fig 2H

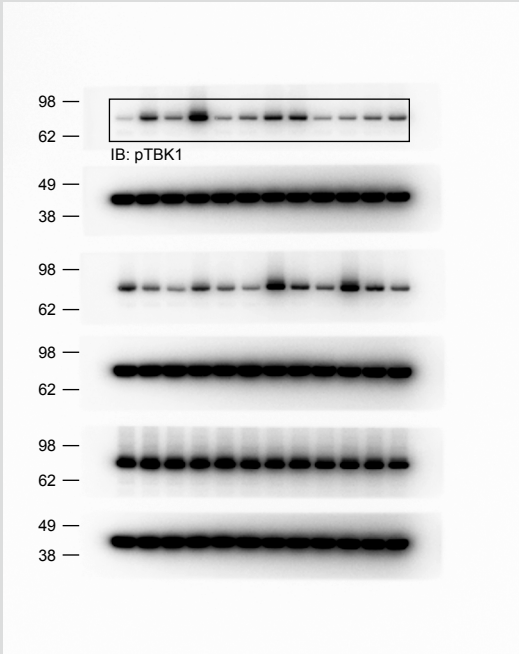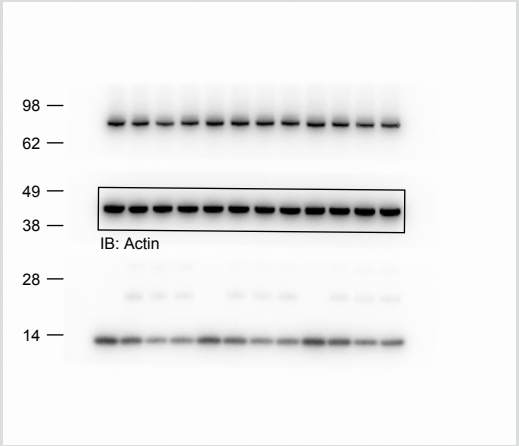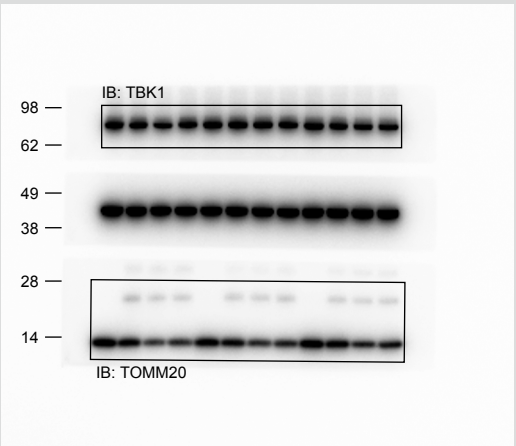

Supplement: Supplementary file 3 — Source Data Fig. 2 [file 44318_2024_36_MOESM3_ESM.zip › Figure2/2H/SourceData_Fig2H.pdf]

Fig 2A

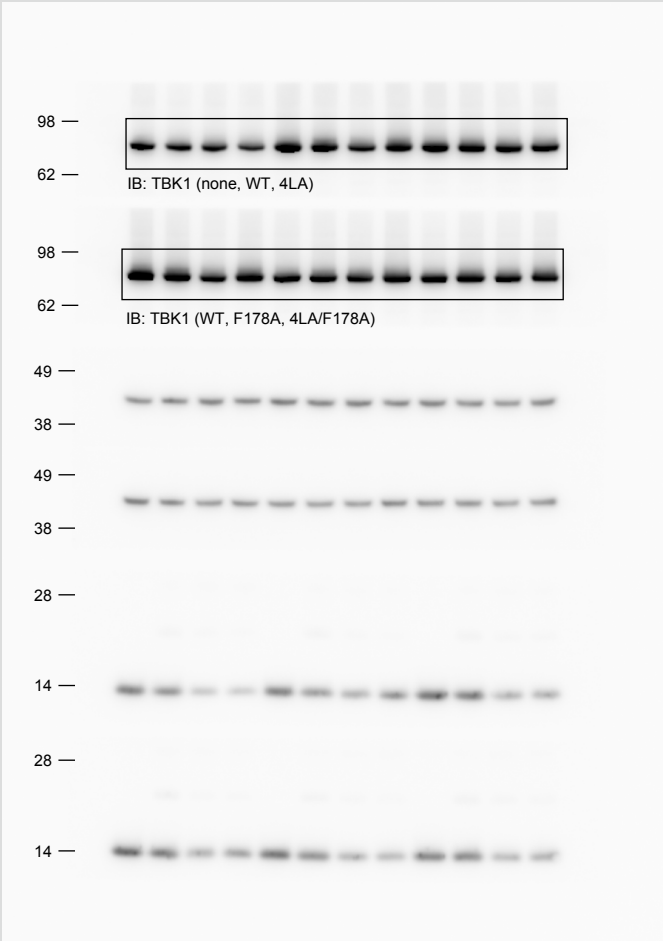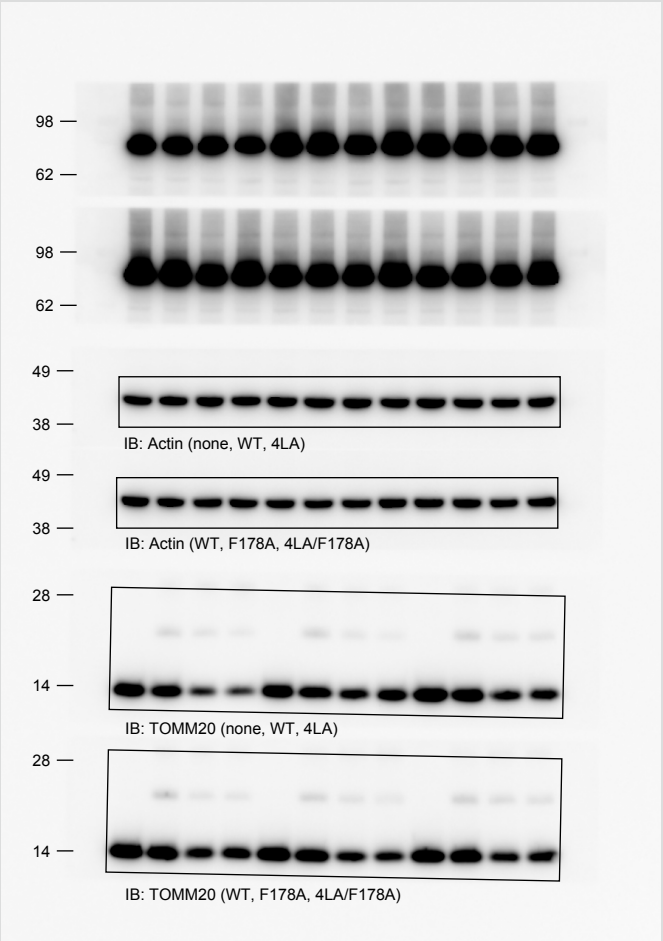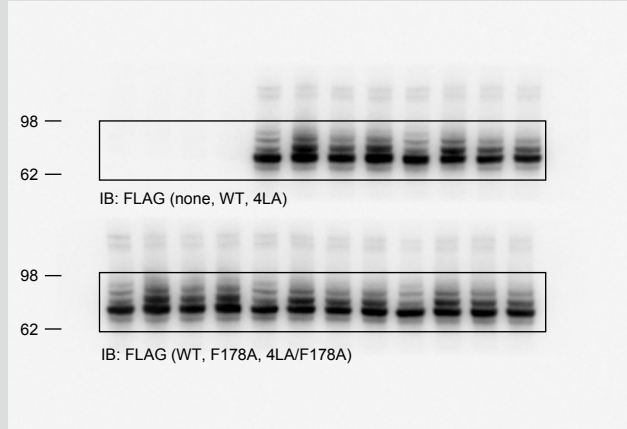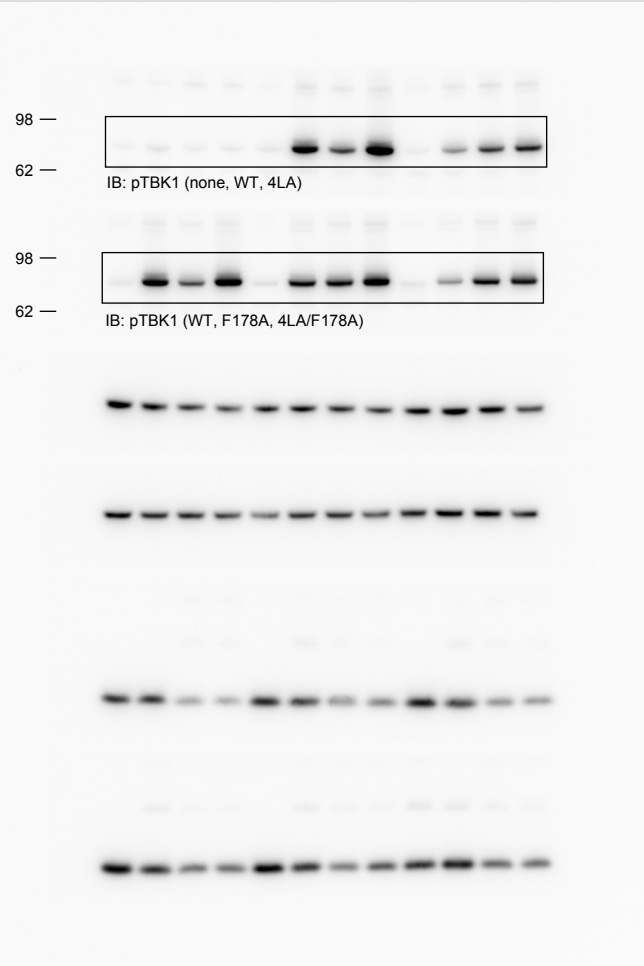

Supplement: Supplementary file 3 — Source Data Fig. 2 [file 44318_2024_36_MOESM3_ESM.zip › Figure2/2A/SourceData_Fig2A.pdf]

Fig 2C

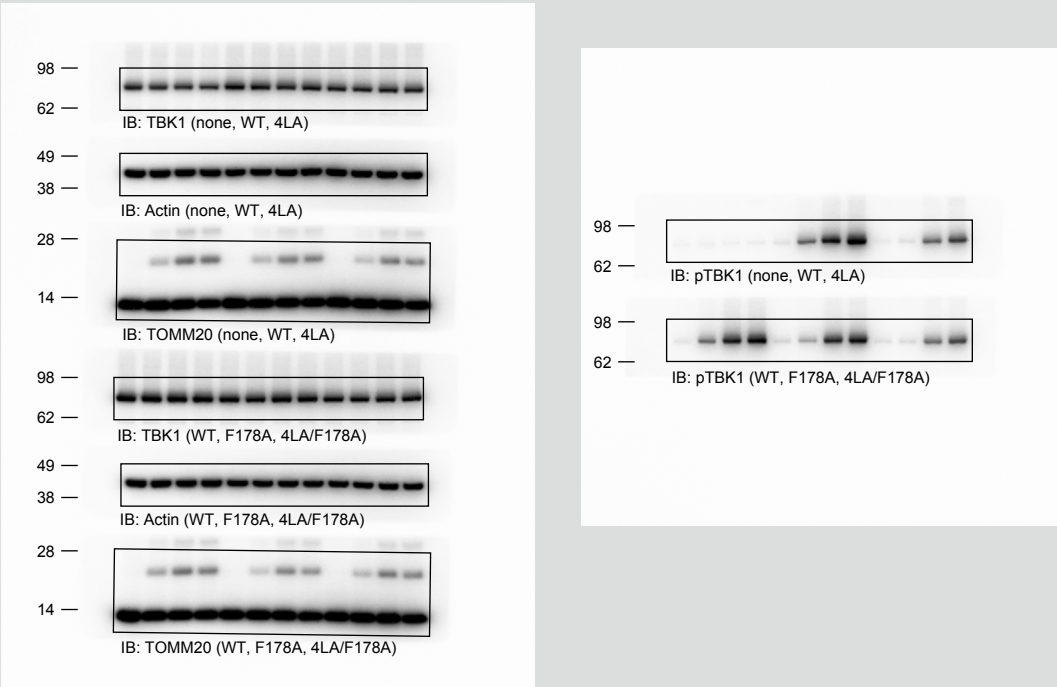

Supplement: Supplementary file 3 — Source Data Fig. 2 [file 44318_2024_36_MOESM3_ESM.zip › Figure2/2C/SourceData_Fig2C.pdf]

Fig 2J

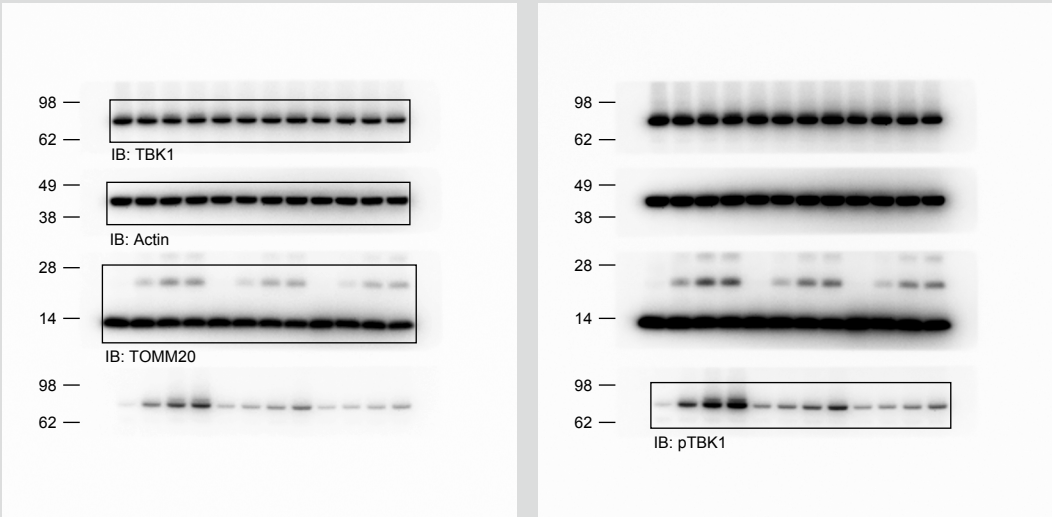

Supplement: Supplementary file 3 — Source Data Fig. 2 [file 44318_2024_36_MOESM3_ESM.zip › Figure2/2J/SourceData_Fig2J.pdf]

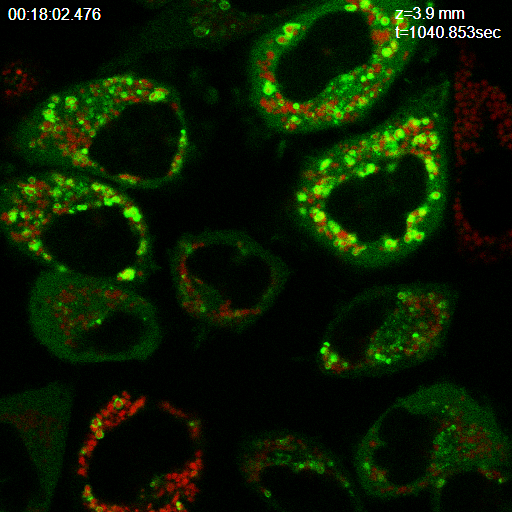

Supplement: Supplementary file 3 — Source Data Fig. 2 [file 44318_2024_36_MOESM3_ESM.zip › Figure2/2L/SourceData_Fig2L_936sec.tif]

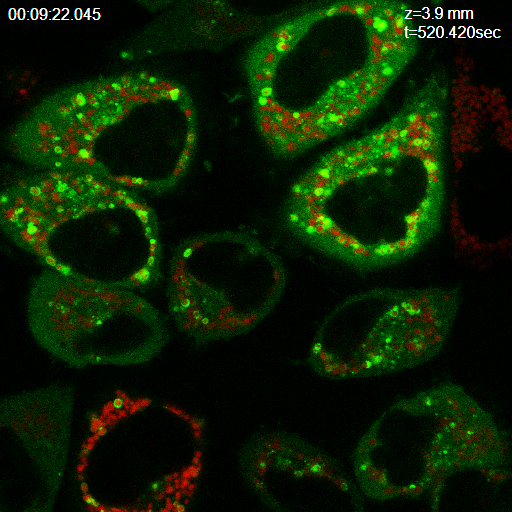

Supplement: Supplementary file 3 — Source Data Fig. 2 [file 44318_2024_36_MOESM3_ESM.zip › Figure2/2L/SourceData_Fig2L_416sec.tif]

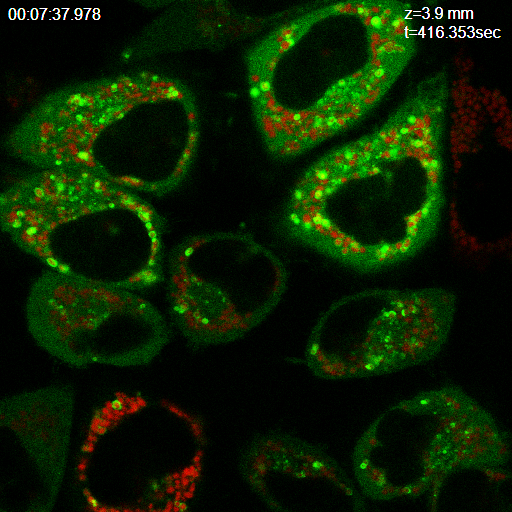

Supplement: Supplementary file 3 — Source Data Fig. 2 [file 44318_2024_36_MOESM3_ESM.zip › Figure2/2L/SourceData_Fig2L_312sec.tif]

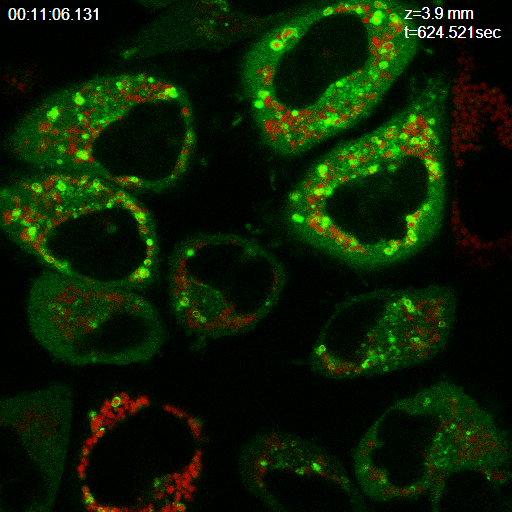

Supplement: Supplementary file 3 — Source Data Fig. 2 [file 44318_2024_36_MOESM3_ESM.zip › Figure2/2L/SourceData_Fig2L_520sec.tif]

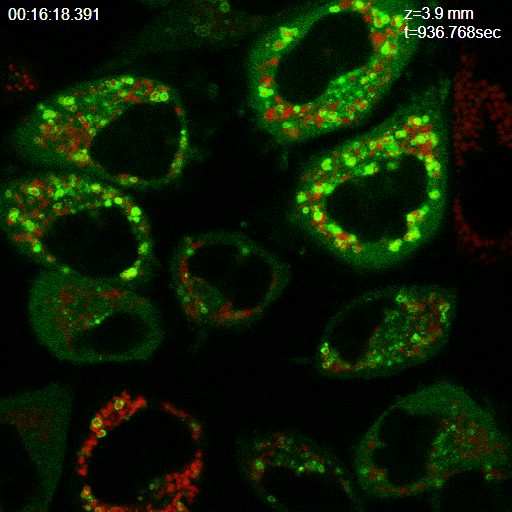

Supplement: Supplementary file 3 — Source Data Fig. 2 [file 44318_2024_36_MOESM3_ESM.zip › Figure2/2L/SourceData_Fig2L_832sec.tif]

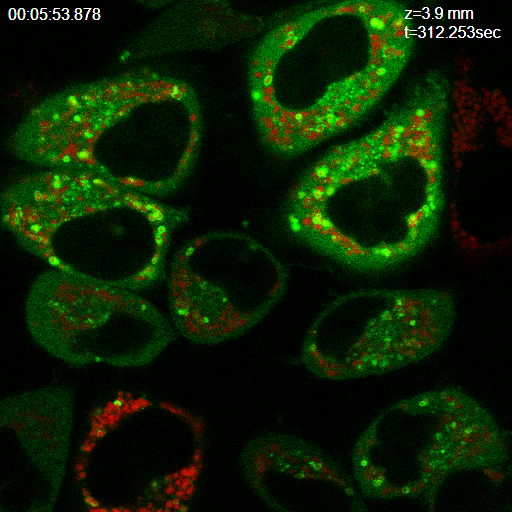

Supplement: Supplementary file 3 — Source Data Fig. 2 [file 44318_2024_36_MOESM3_ESM.zip › Figure2/2L/SourceData_Fig2L_208sec.tif]

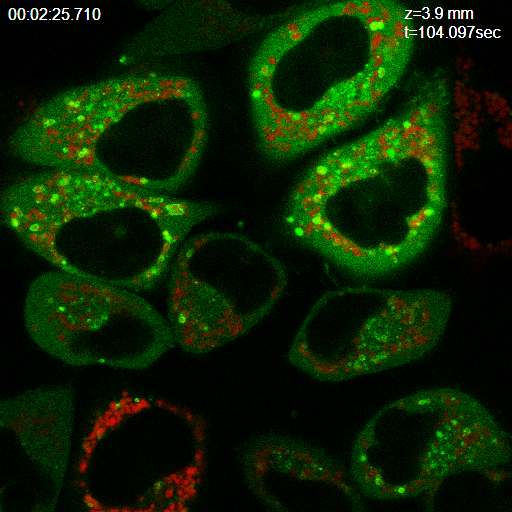

Supplement: Supplementary file 3 — Source Data Fig. 2 [file 44318_2024_36_MOESM3_ESM.zip › Figure2/2L/SourceData_Fig2L_0sec.tif]

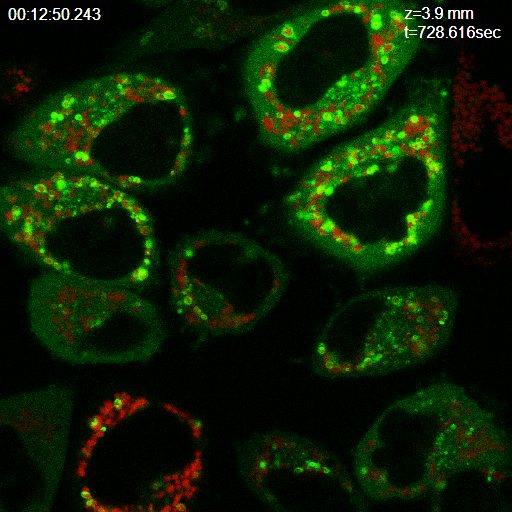

Supplement: Supplementary file 3 — Source Data Fig. 2 [file 44318_2024_36_MOESM3_ESM.zip › Figure2/2L/SourceData_Fig2L_624sec.tif]

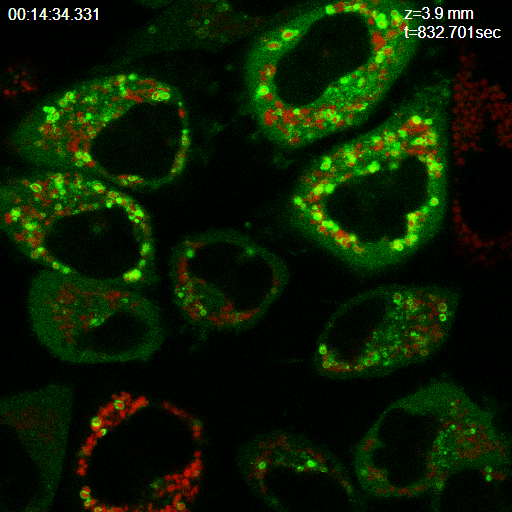

Supplement: Supplementary file 3 — Source Data Fig. 2 [file 44318_2024_36_MOESM3_ESM.zip › Figure2/2L/SourceData_Fig2L_728sec.tif]

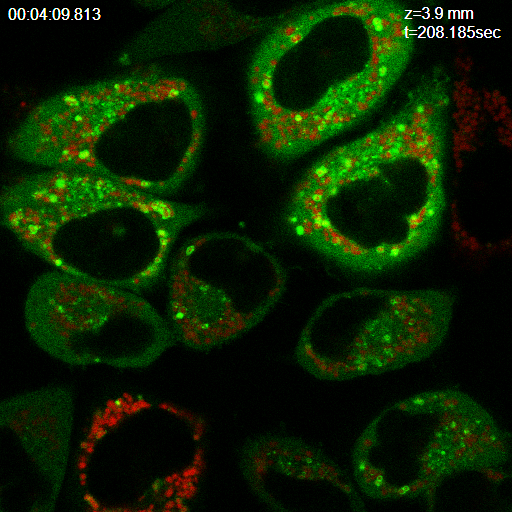

Supplement: Supplementary file 3 — Source Data Fig. 2 [file 44318_2024_36_MOESM3_ESM.zip › Figure2/2L/SourceData_Fig2L_104sec.tif]

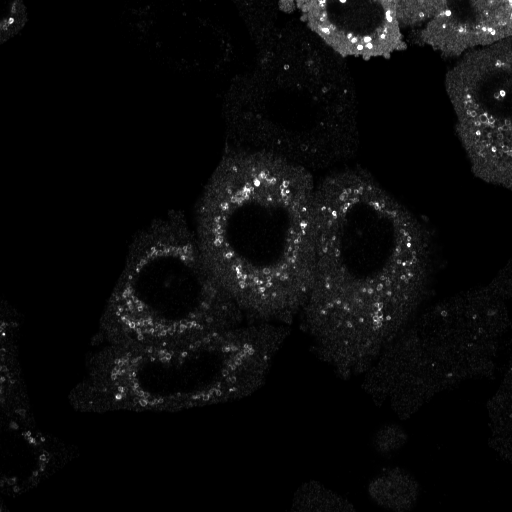

Supplement: Supplementary file 3 — Source Data Fig. 2 [file 44318_2024_36_MOESM3_ESM.zip › Figure2/2E/SourceData_Fig2E_OPTN_4LA_F178A.tif]

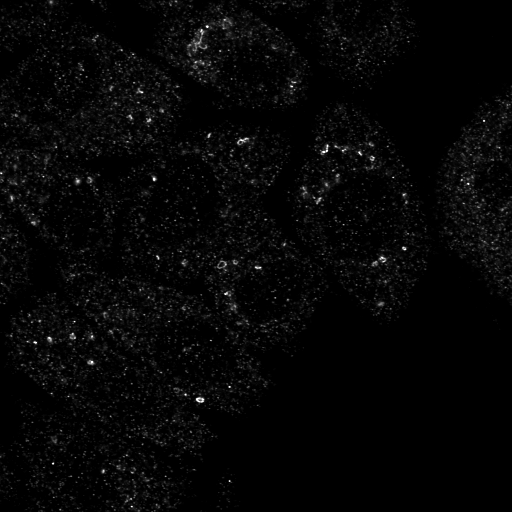

Supplement: Supplementary file 3 — Source Data Fig. 2 [file 44318_2024_36_MOESM3_ESM.zip › Figure2/2E/SourceData_Fig2E_OPTN_WT_WIPI2.tif]

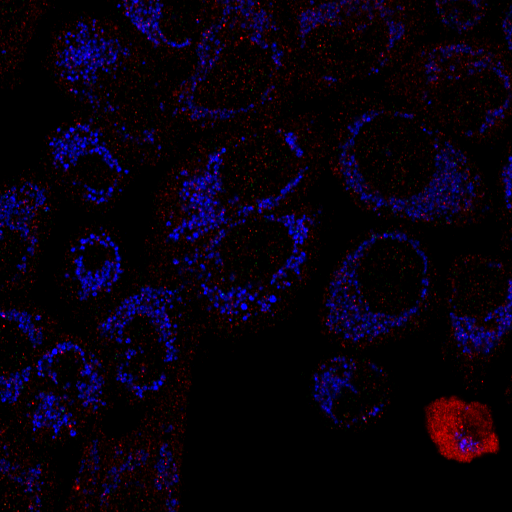

Supplement: Supplementary file 3 — Source Data Fig. 2 [file 44318_2024_36_MOESM3_ESM.zip › Figure2/2E/SourceData_Fig2E_none_Merge.tif]

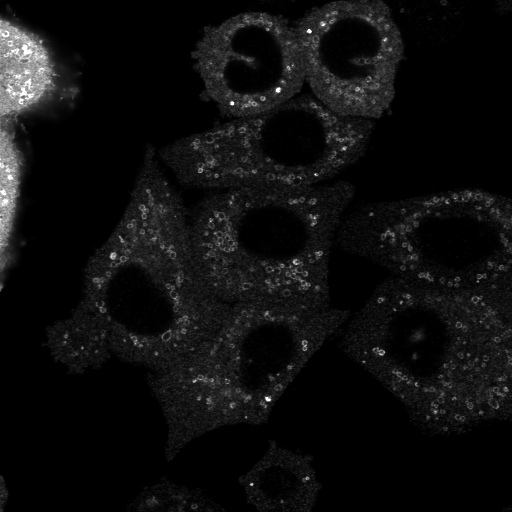

Supplement: Supplementary file 3 — Source Data Fig. 2 [file 44318_2024_36_MOESM3_ESM.zip › Figure2/2E/SourceData_Fig2E_OPTN_4LA.tif]

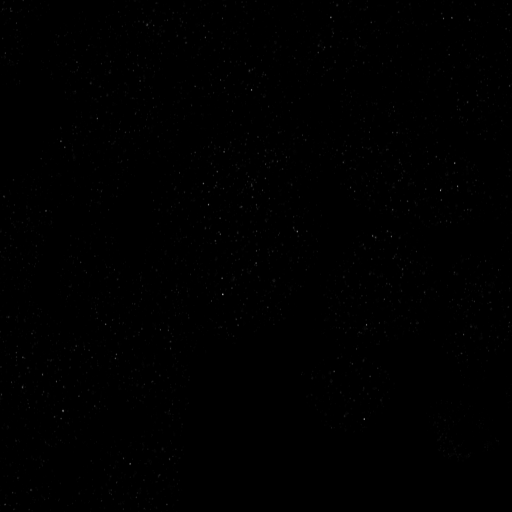

Supplement: Supplementary file 3 — Source Data Fig. 2 [file 44318_2024_36_MOESM3_ESM.zip › Figure2/2E/SourceData_Fig2E_none.tif]

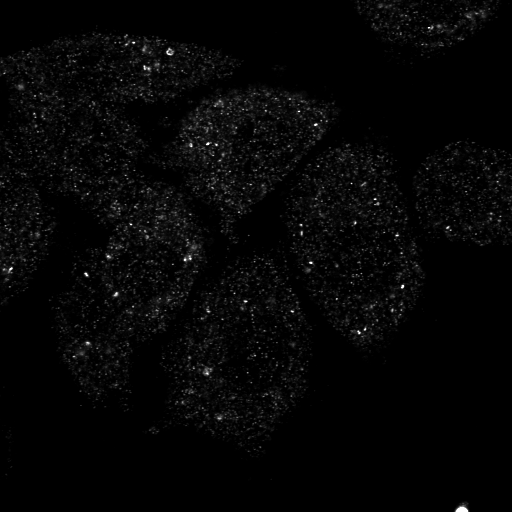

Supplement: Supplementary file 3 — Source Data Fig. 2 [file 44318_2024_36_MOESM3_ESM.zip › Figure2/2E/SourceData_Fig2E_OPTN_F178A_WIPI2.tif]

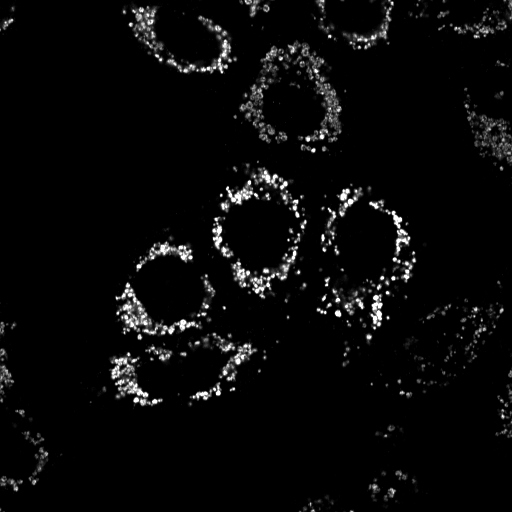

Supplement: Supplementary file 3 — Source Data Fig. 2 [file 44318_2024_36_MOESM3_ESM.zip › Figure2/2E/SourceData_Fig2E_OPTN_4LA_F178A_HSP60.tif]

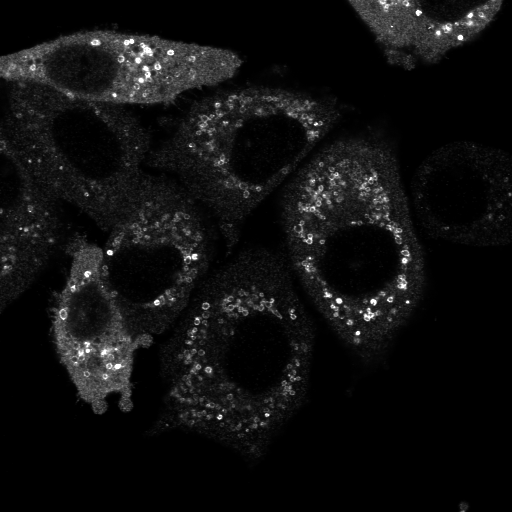

Supplement: Supplementary file 3 — Source Data Fig. 2 [file 44318_2024_36_MOESM3_ESM.zip › Figure2/2E/SourceData_Fig2E_OPTN_F178A.tif]

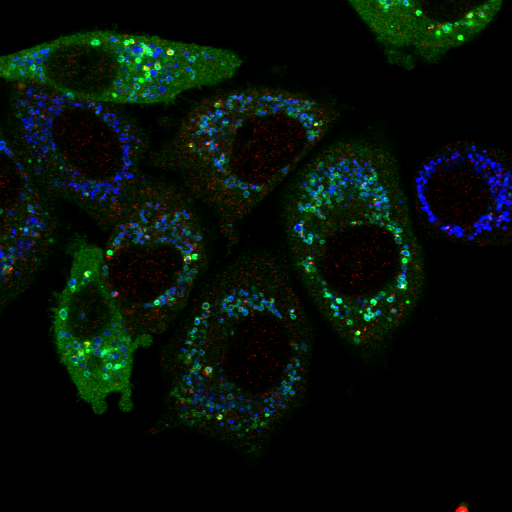

Supplement: Supplementary file 3 — Source Data Fig. 2 [file 44318_2024_36_MOESM3_ESM.zip › Figure2/2E/SourceData_Fig2E_OPTN_F178A_Merge.tif]

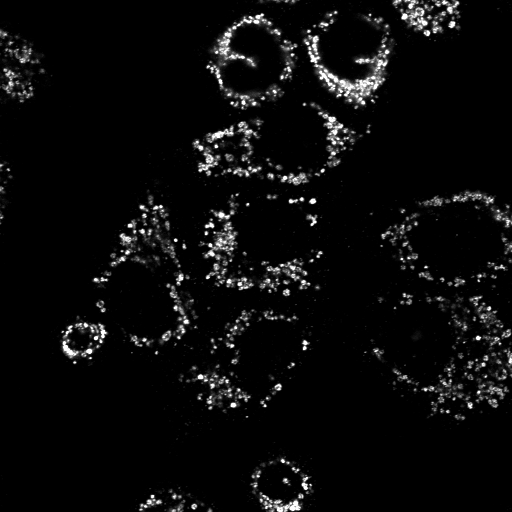

Supplement: Supplementary file 3 — Source Data Fig. 2 [file 44318_2024_36_MOESM3_ESM.zip › Figure2/2E/SourceData_Fig2E_OPTN_4LA_HSP60.tif]

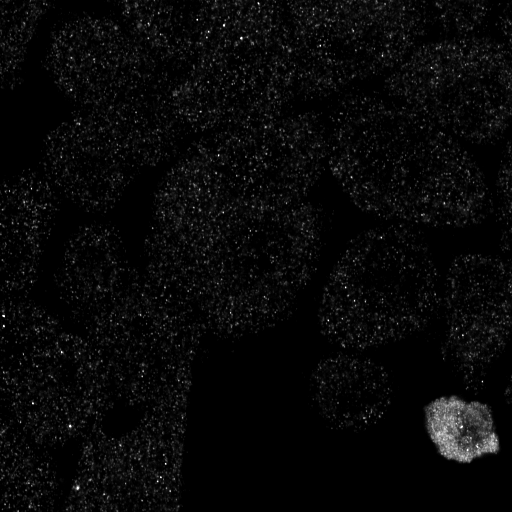

Supplement: Supplementary file 3 — Source Data Fig. 2 [file 44318_2024_36_MOESM3_ESM.zip › Figure2/2E/SourceData_Fig2E_none_WIPI2.tif]

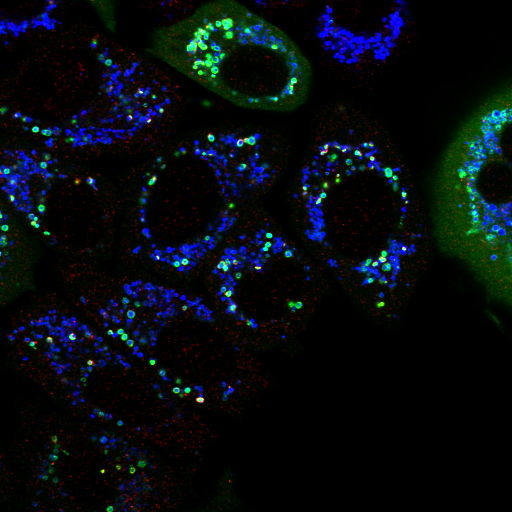

Supplement: Supplementary file 3 — Source Data Fig. 2 [file 44318_2024_36_MOESM3_ESM.zip › Figure2/2E/SourceData_Fig2E_OPTN_WT_Merge.tif]

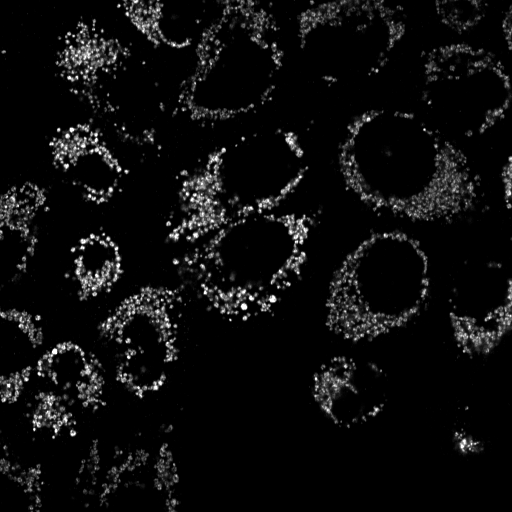

Supplement: Supplementary file 3 — Source Data Fig. 2 [file 44318_2024_36_MOESM3_ESM.zip › Figure2/2E/SourceData_Fig2E_none_HSP60.tif]

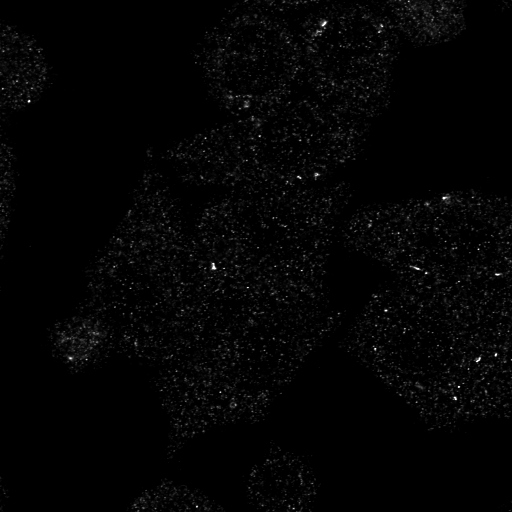

Supplement: Supplementary file 3 — Source Data Fig. 2 [file 44318_2024_36_MOESM3_ESM.zip › Figure2/2E/SourceData_Fig2E_OPTN_4LA_WIPI2.tif]

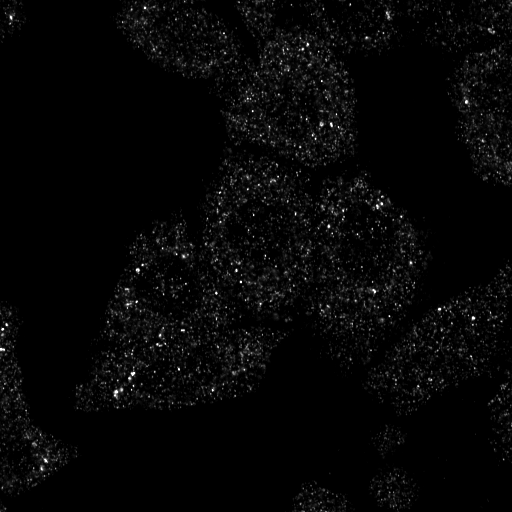

Supplement: Supplementary file 3 — Source Data Fig. 2 [file 44318_2024_36_MOESM3_ESM.zip › Figure2/2E/SourceData_Fig2E_OPTN_4LA_F178A_WIPI2.tif]

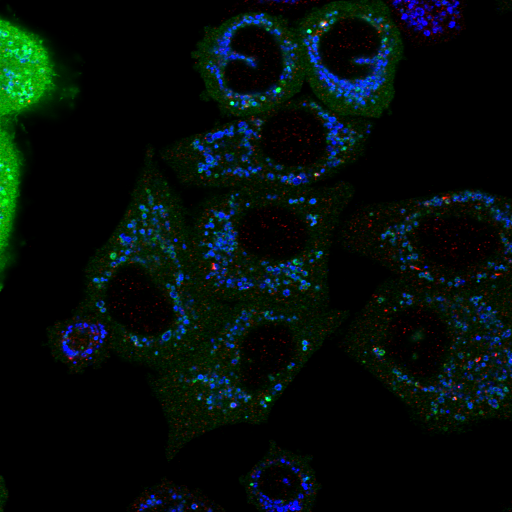

Supplement: Supplementary file 3 — Source Data Fig. 2 [file 44318_2024_36_MOESM3_ESM.zip › Figure2/2E/SourceData_Fig2E_OPTN_4LA_Merge.tif]

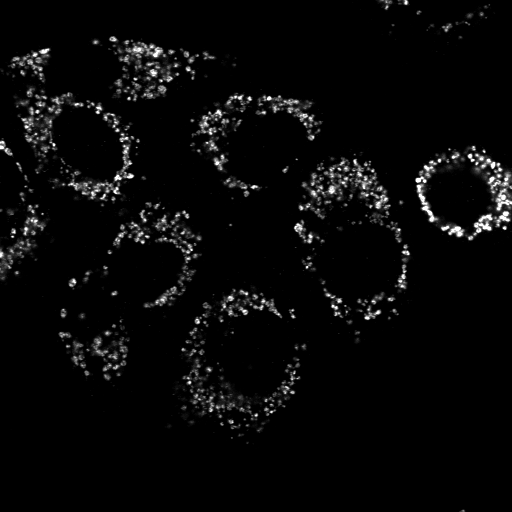

Supplement: Supplementary file 3 — Source Data Fig. 2 [file 44318_2024_36_MOESM3_ESM.zip › Figure2/2E/SourceData_Fig2E_OPTN_F178A_HSP60.tif]

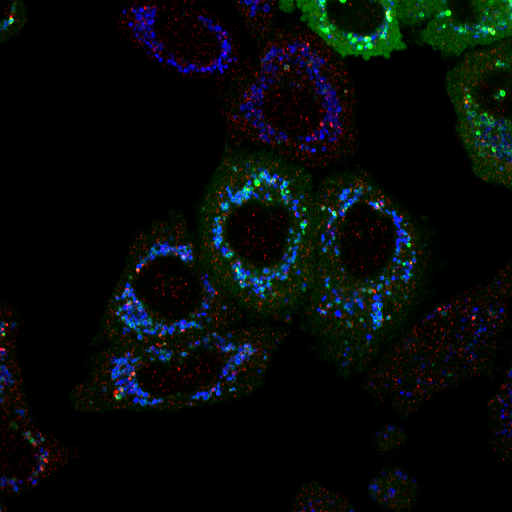

Supplement: Supplementary file 3 — Source Data Fig. 2 [file 44318_2024_36_MOESM3_ESM.zip › Figure2/2E/SourceData_Fig2E_OPTN_4LA_F178A_Merge.tif]

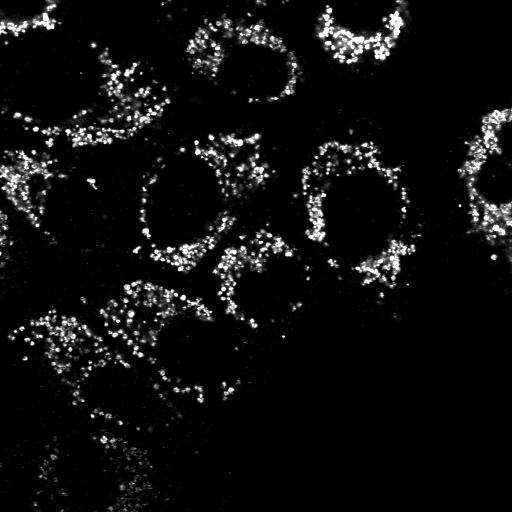

Supplement: Supplementary file 3 — Source Data Fig. 2 [file 44318_2024_36_MOESM3_ESM.zip › Figure2/2E/SourceData_Fig2E_OPTN_WT_HSP60.tif]

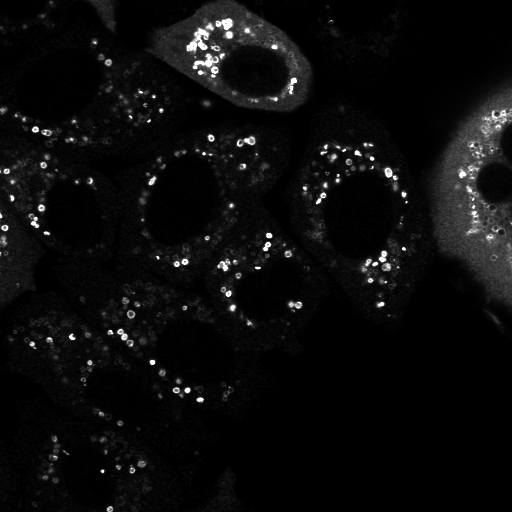

Supplement: Supplementary file 3 — Source Data Fig. 2 [file 44318_2024_36_MOESM3_ESM.zip › Figure2/2E/SourceData_Fig2E_OPTN_WT.tif]

Fig 3B

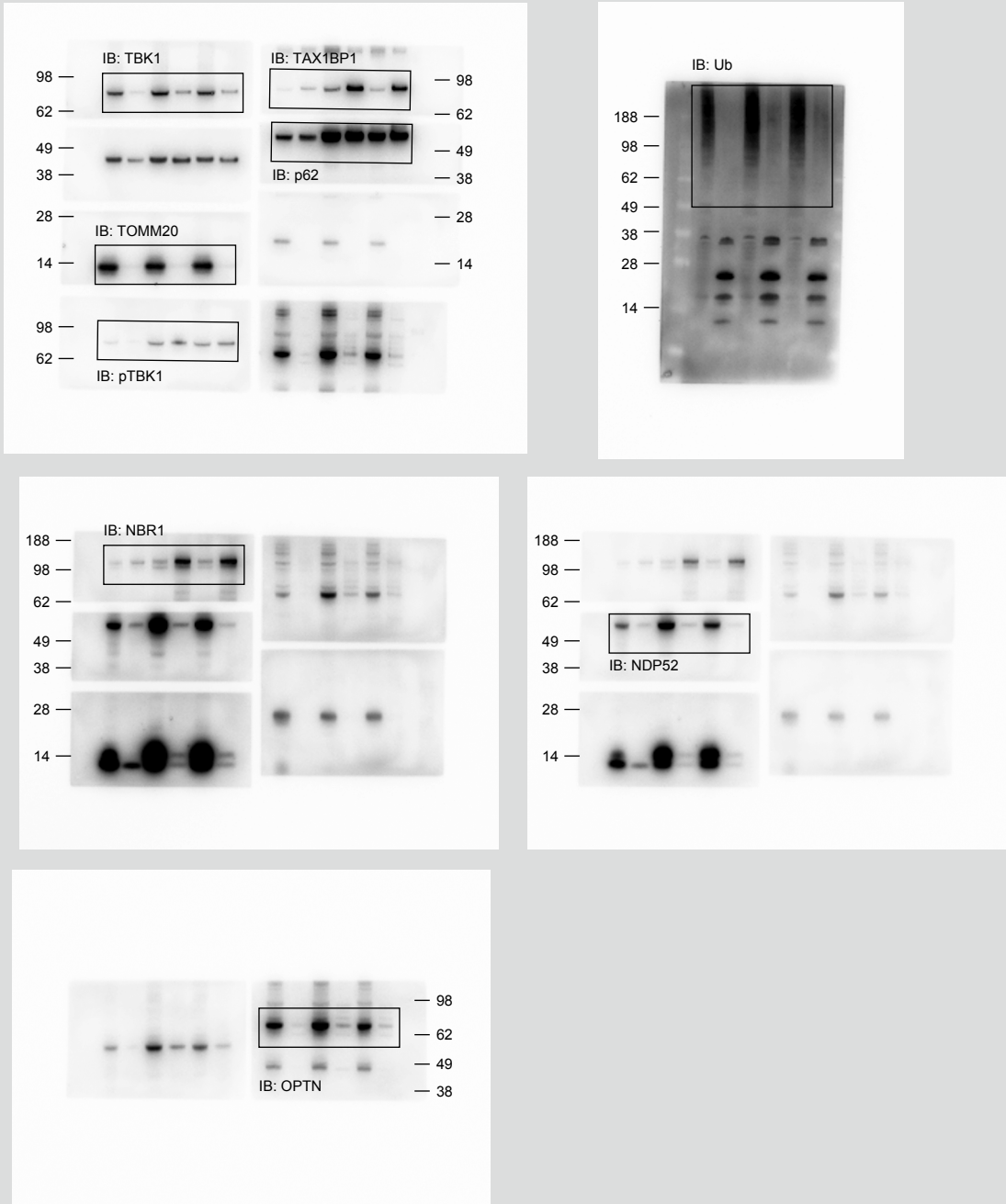

Supplement: Supplementary file 4 — Source Data Fig. 3 [file 44318_2024_36_MOESM4_ESM.zip › Figure3/3B/SourceData_Fig3B.pdf]

Fig 3D

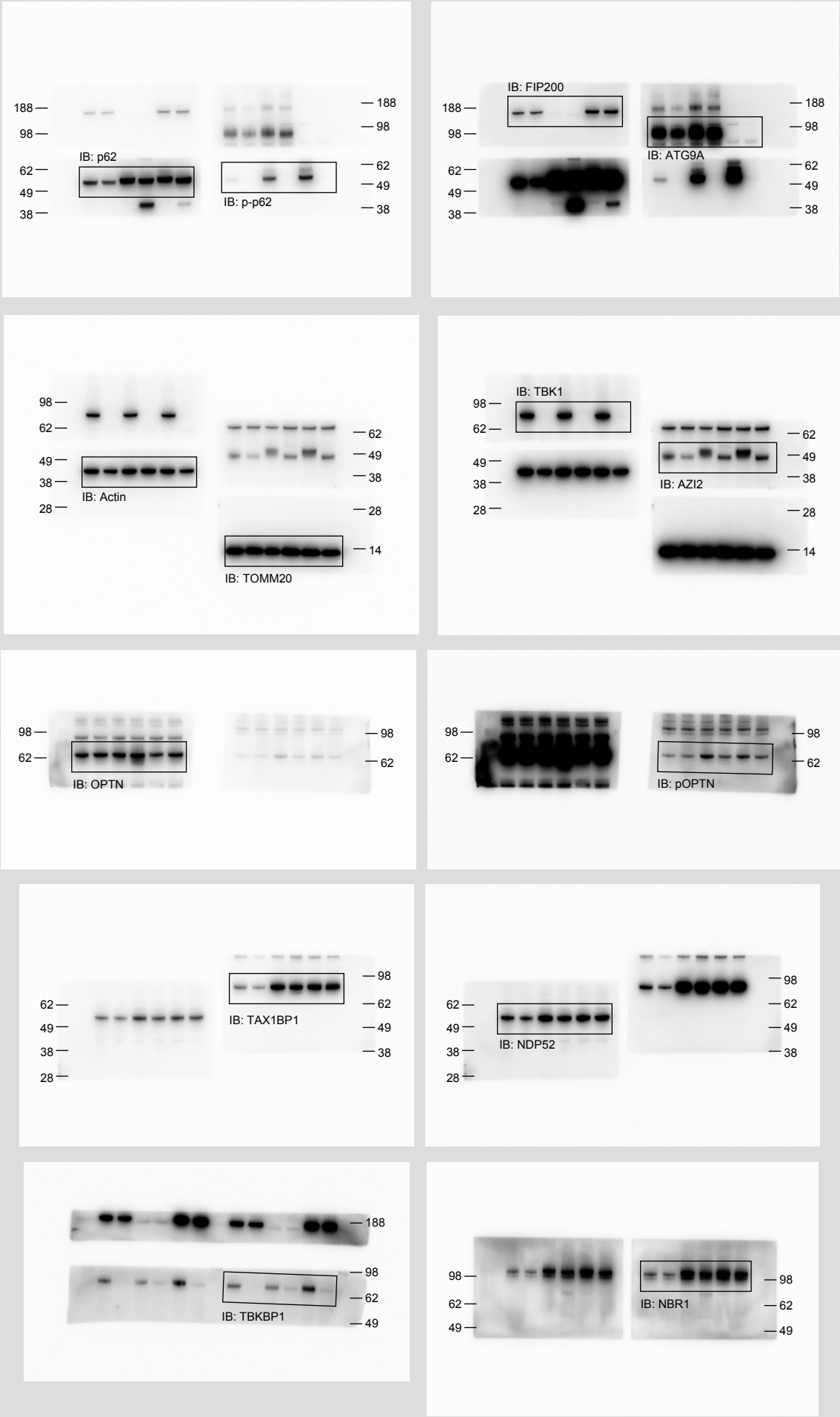

Supplement: Supplementary file 4 — Source Data Fig. 3 [file 44318_2024_36_MOESM4_ESM.zip › Figure3/3D/SourceData_Fig3D.pdf]

Fig 3E

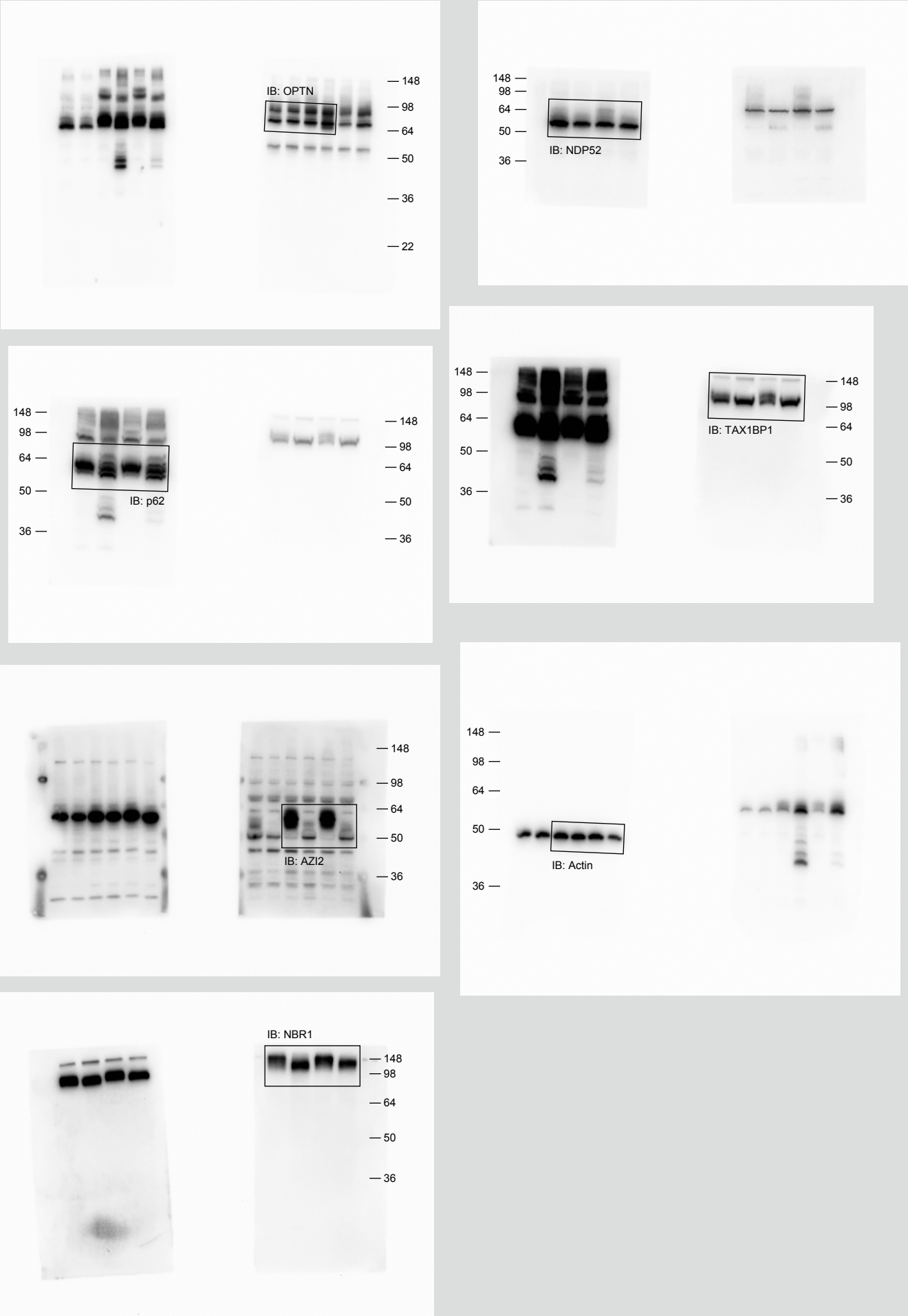

Supplement: Supplementary file 4 — Source Data Fig. 3 [file 44318_2024_36_MOESM4_ESM.zip › Figure3/3E/SourceData_Fig3E.pdf]

Fig 3F

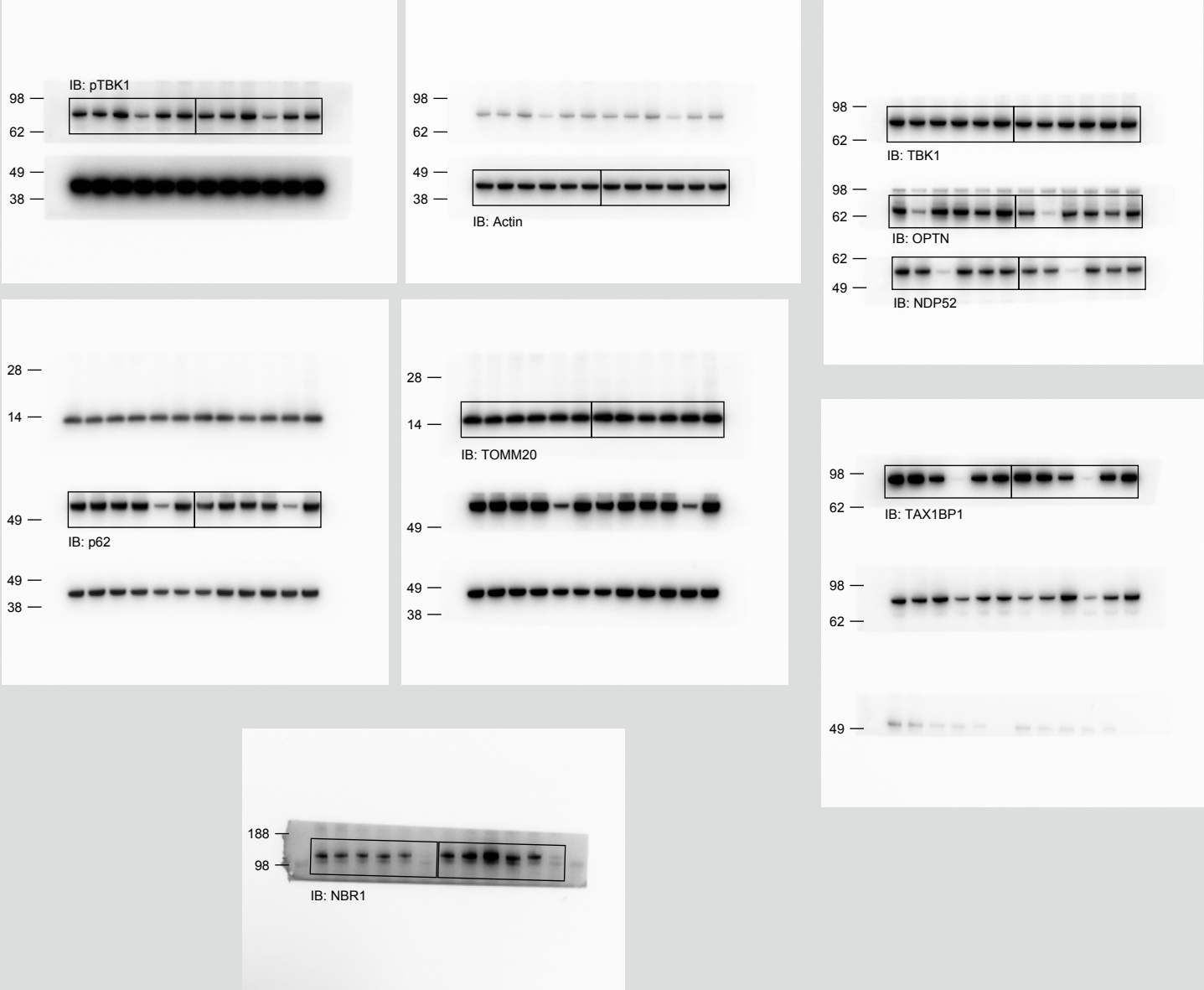

Supplement: Supplementary file 4 — Source Data Fig. 3 [file 44318_2024_36_MOESM4_ESM.zip › Figure3/3F/SourceData_Fig3F.pdf]

Fig 4B

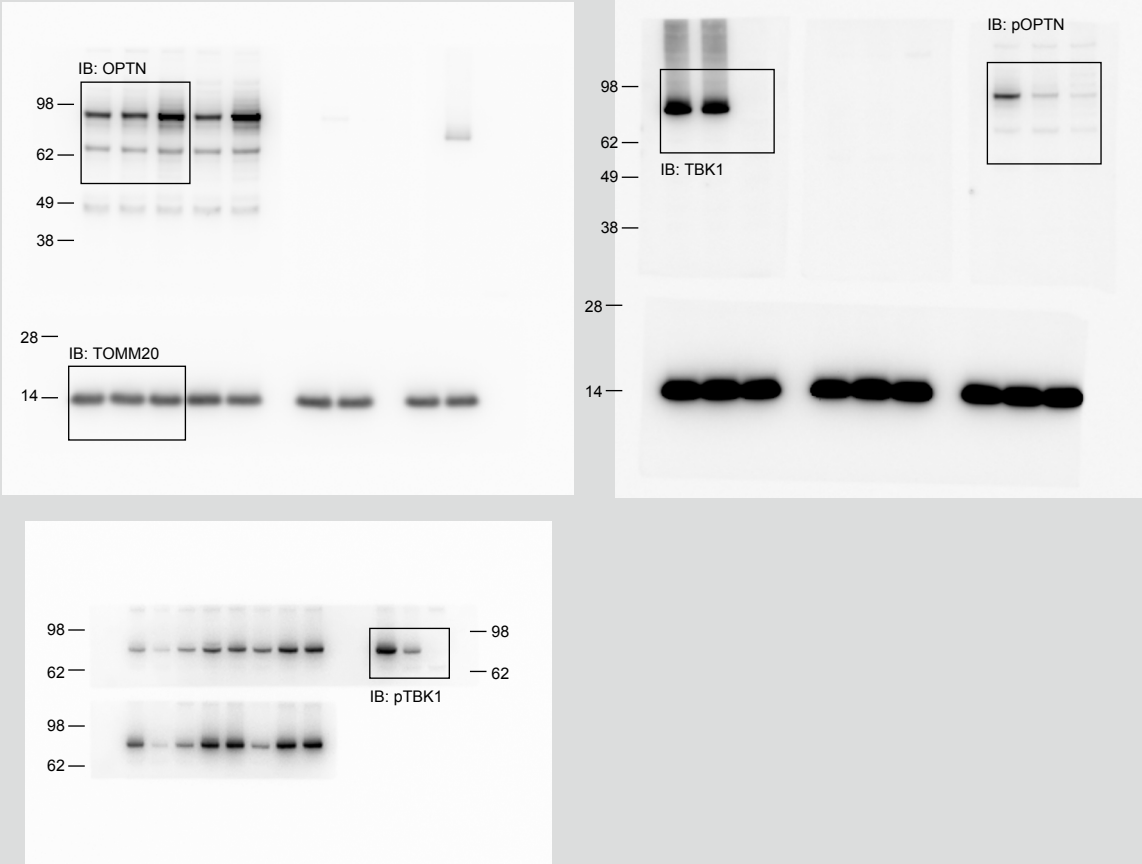

Supplement: Supplementary file 5 — Source Data Fig. 4 [file 44318_2024_36_MOESM5_ESM.zip › Figure4/4B/SourceData_Fig4B.pdf]

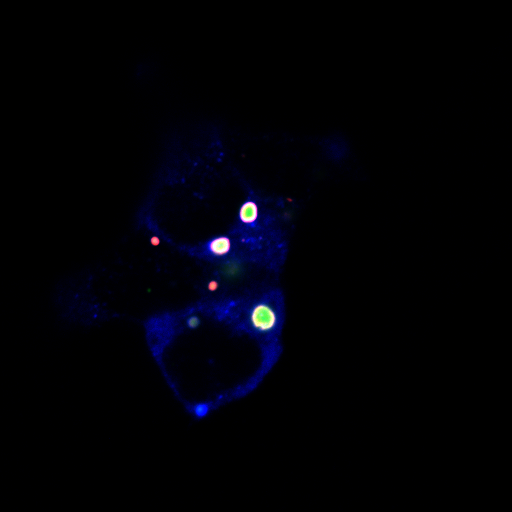

Supplement: Supplementary file 5 — Source Data Fig. 4 [file 44318_2024_36_MOESM5_ESM.zip › Figure4/4C/SourceData_Fig4C_WT.tif]

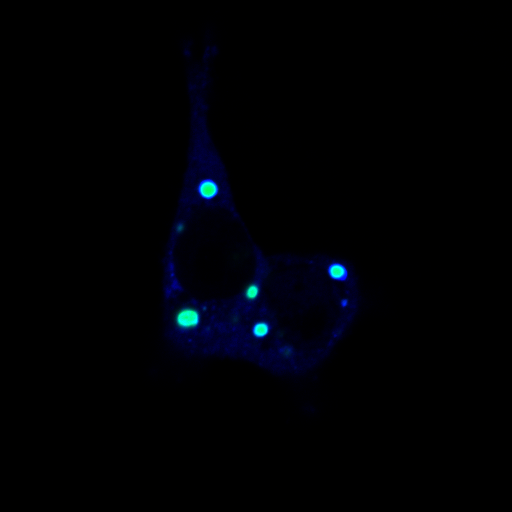

Supplement: Supplementary file 5 — Source Data Fig. 4 [file 44318_2024_36_MOESM5_ESM.zip › Figure4/4C/SourceData_Fig4C_TBK1KO.tif]

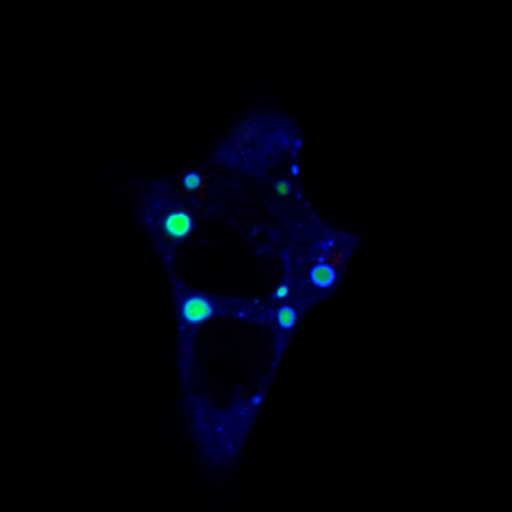

Supplement: Supplementary file 5 — Source Data Fig. 4 [file 44318_2024_36_MOESM5_ESM.zip › Figure4/4D/SourceData_Fig4D_TBK1KO.tif]

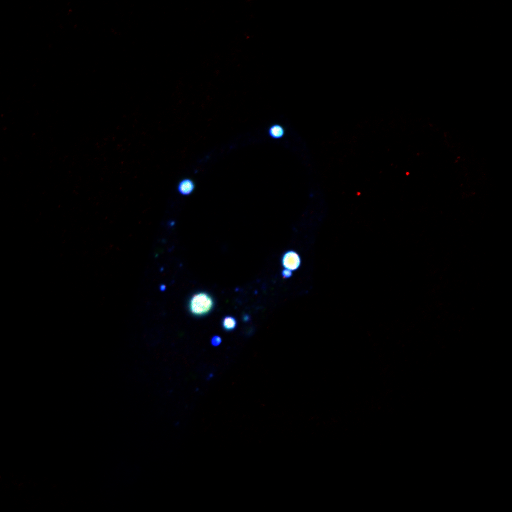

Supplement: Supplementary file 5 — Source Data Fig. 4 [file 44318_2024_36_MOESM5_ESM.zip › Figure4/4D/SourceData_Fig4D_WT.tif]

Fig 5A

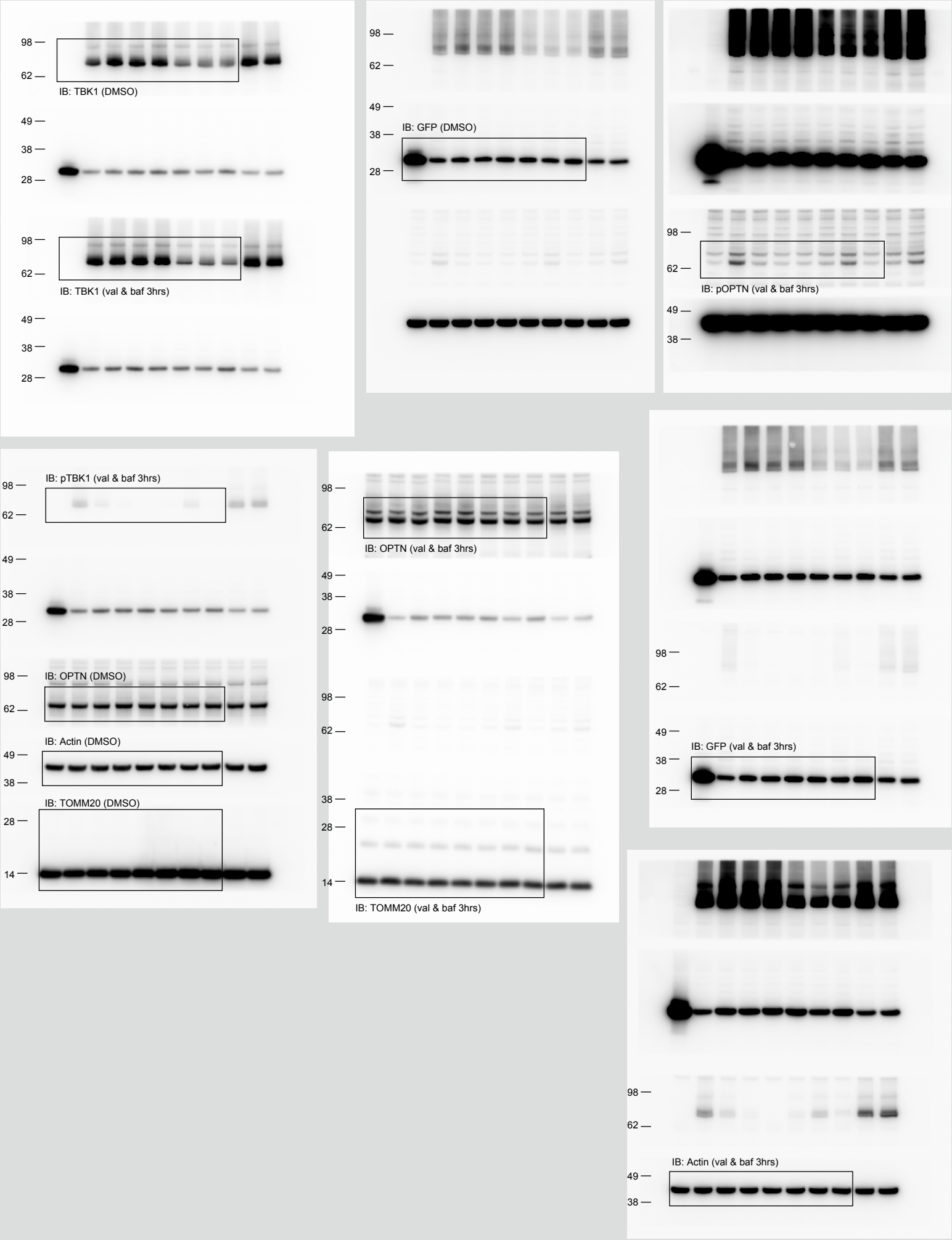

Supplement: Supplementary file 6 — Source Data Fig. 5 [file 44318_2024_36_MOESM6_ESM.zip › Figure5/5A/SourceData_Fig5A.pdf]

Fig 5E

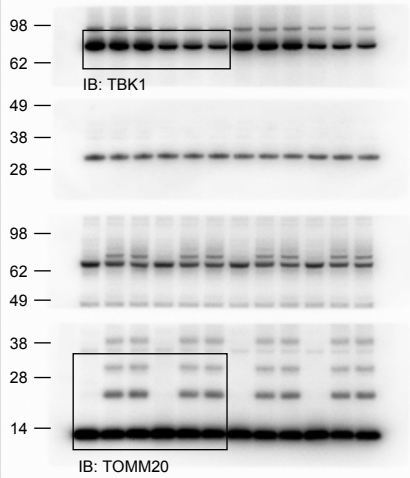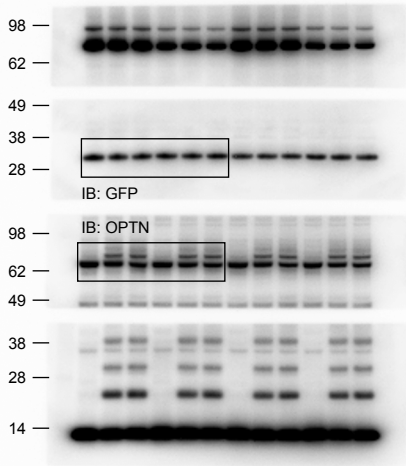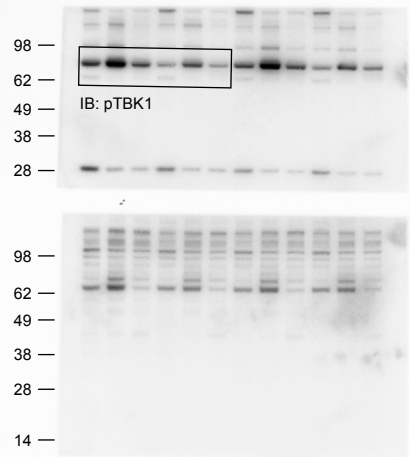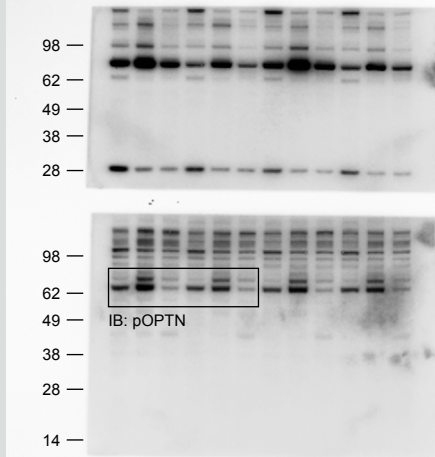

Supplement: Supplementary file 6 — Source Data Fig. 5 [file 44318_2024_36_MOESM6_ESM.zip › Figure5/5E/SourceData_Fig5E.pdf]

Fig 6F

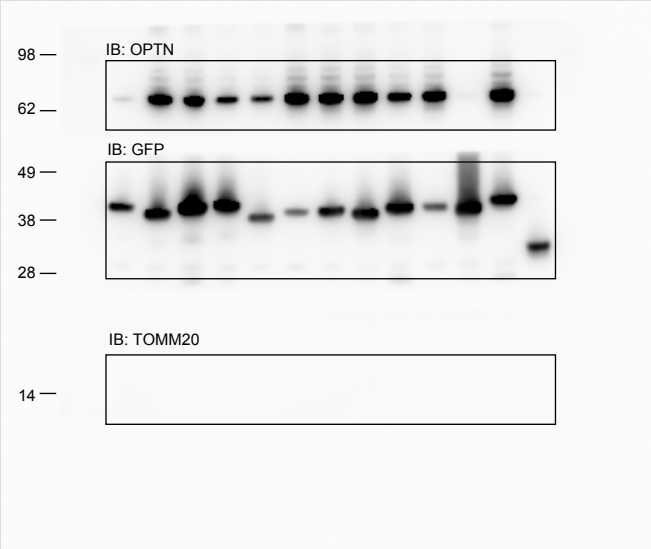

Supplement: Supplementary file 7 — Source Data Fig. 6 [file 44318_2024_36_MOESM7_ESM.zip › Figure6/6F/SourceData_Fig6F.pdf]

Fig 6H

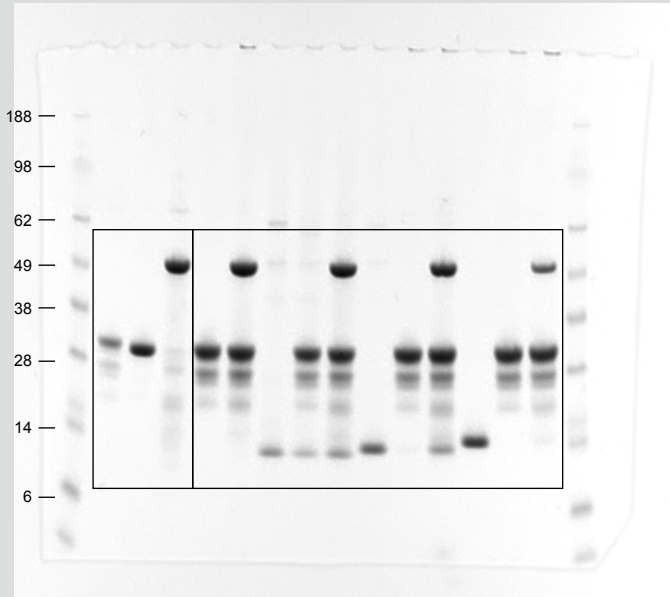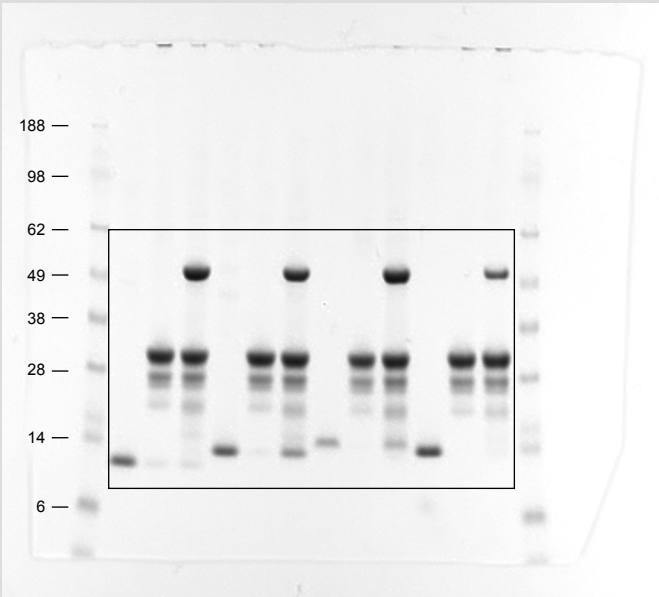

Supplement: Supplementary file 7 — Source Data Fig. 6 [file 44318_2024_36_MOESM7_ESM.zip › Figure6/6H/SourceData_Fig6H.pdf]

Fig 6G

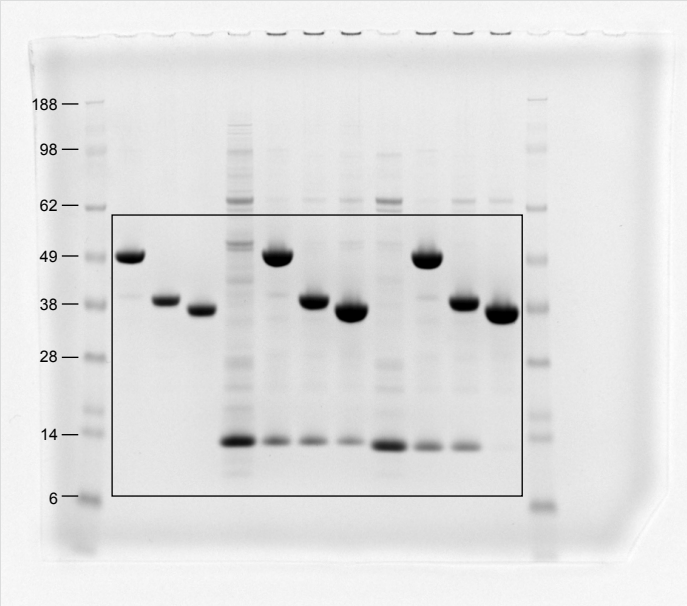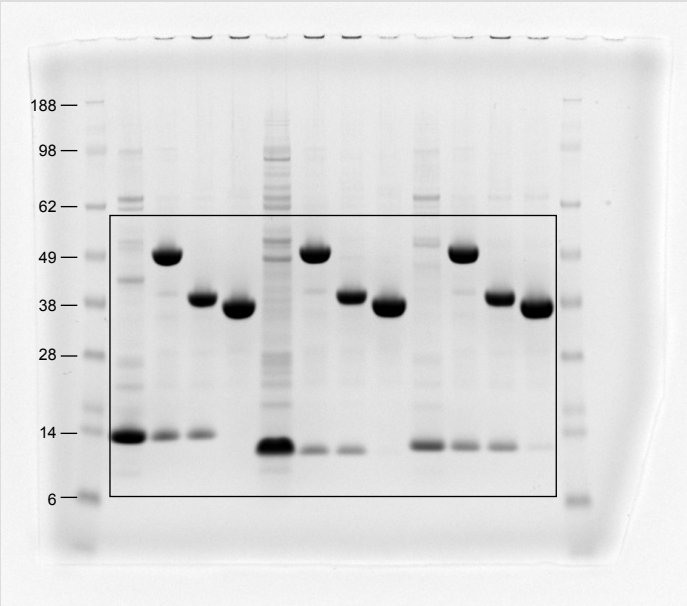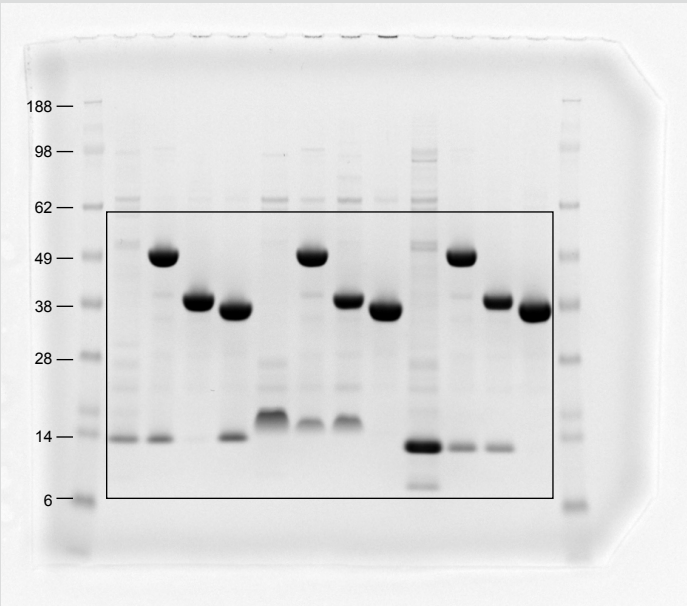

Supplement: Supplementary file 7 — Source Data Fig. 6 [file 44318_2024_36_MOESM7_ESM.zip › Figure6/6G/SourceData_Fig6G.pdf]

Fig 6E

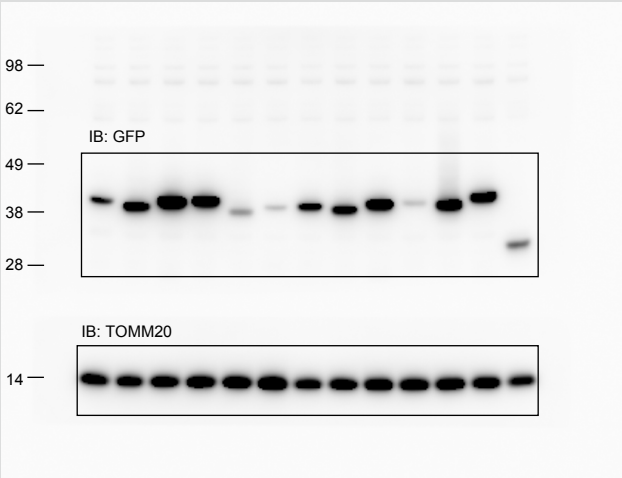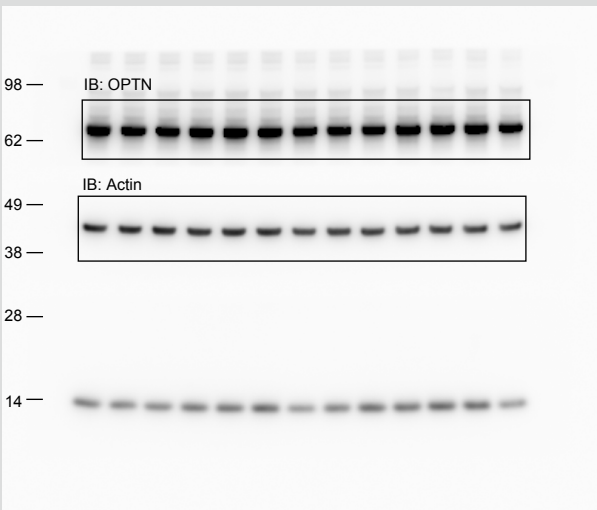

Supplement: Supplementary file 7 — Source Data Fig. 6 [file 44318_2024_36_MOESM7_ESM.zip › Figure6/6E/SourceData_Fig6E.pdf]

Fig 7B

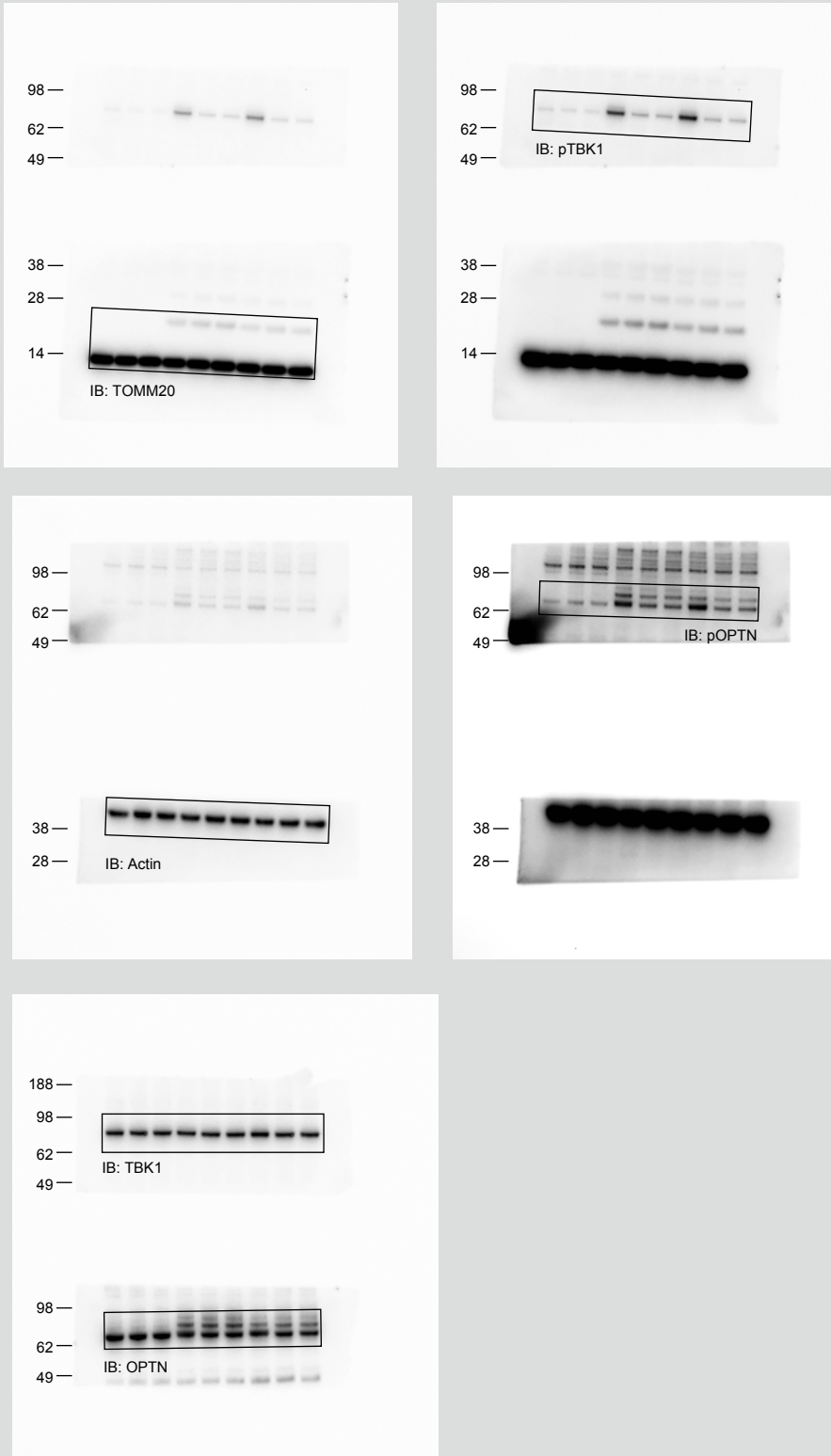

Supplement: Supplementary file 8 — Source Data Fig. 7 [file 44318_2024_36_MOESM8_ESM.zip › Figure7/7B/SourceData_Fig7B.pdf]

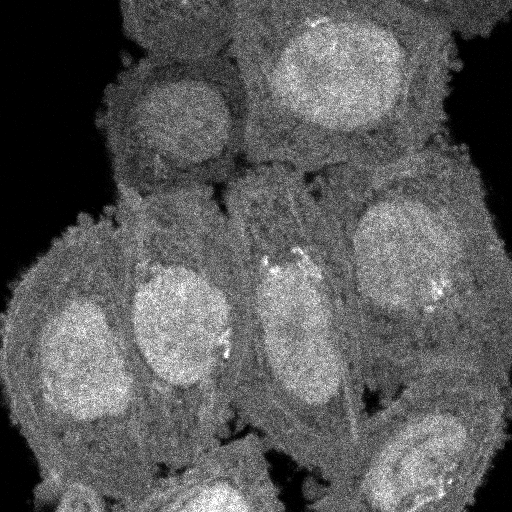

Supplement: Supplementary file 8 — Source Data Fig. 7 [file 44318_2024_36_MOESM8_ESM.zip › Figure7/7A/SourceData_Fig7A_MonoB3_NT_GFP.tif]

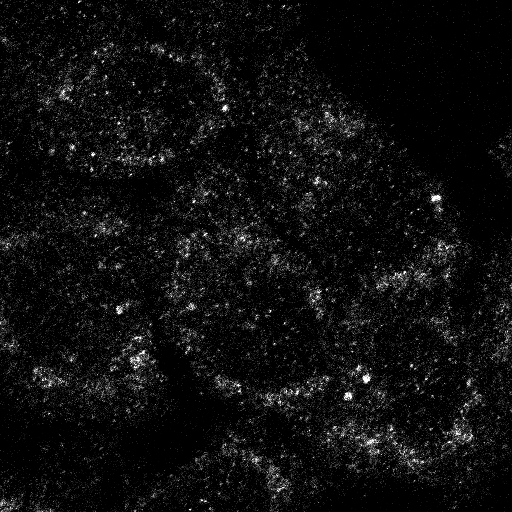

Supplement: Supplementary file 8 — Source Data Fig. 7 [file 44318_2024_36_MOESM8_ESM.zip › Figure7/7A/SourceData_Fig7A_MonoB4_valbaf90min_OPTN.tif]

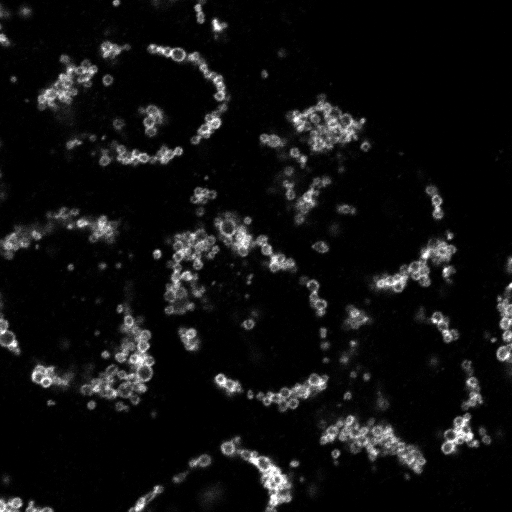

Supplement: Supplementary file 8 — Source Data Fig. 7 [file 44318_2024_36_MOESM8_ESM.zip › Figure7/7A/SourceData_Fig7A_MonoB4_valbaf90min_TOMM20.tif]

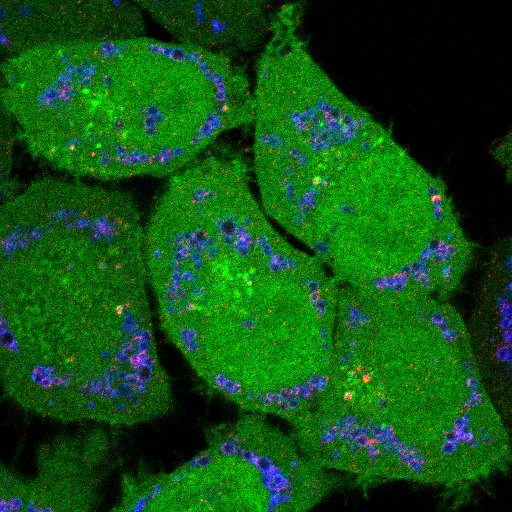

Supplement: Supplementary file 8 — Source Data Fig. 7 [file 44318_2024_36_MOESM8_ESM.zip › Figure7/7A/SourceData_Fig7A_MonoB4_valbaf90min_Merge.tif]

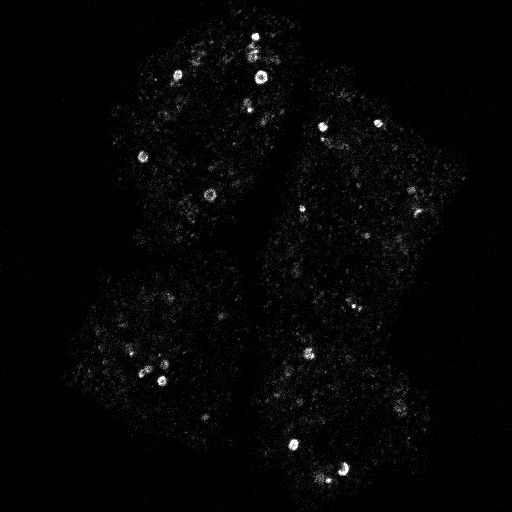

Supplement: Supplementary file 8 — Source Data Fig. 7 [file 44318_2024_36_MOESM8_ESM.zip › Figure7/7A/SourceData_Fig7A_GFP_valbaf90min_OPTN.tif]

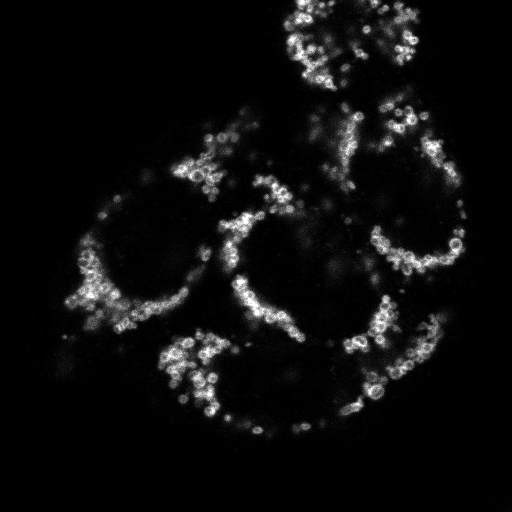

Supplement: Supplementary file 8 — Source Data Fig. 7 [file 44318_2024_36_MOESM8_ESM.zip › Figure7/7A/SourceData_Fig7A_MonoB3_valbaf90min_TOMM20.tif]

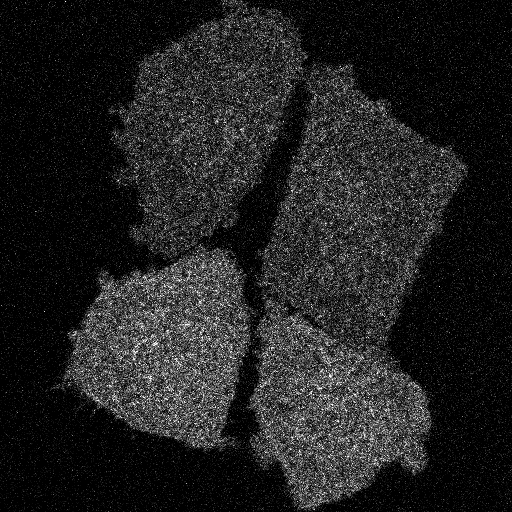

Supplement: Supplementary file 8 — Source Data Fig. 7 [file 44318_2024_36_MOESM8_ESM.zip › Figure7/7A/SourceData_Fig7A_GFP_valbaf90min_GFP.tif]

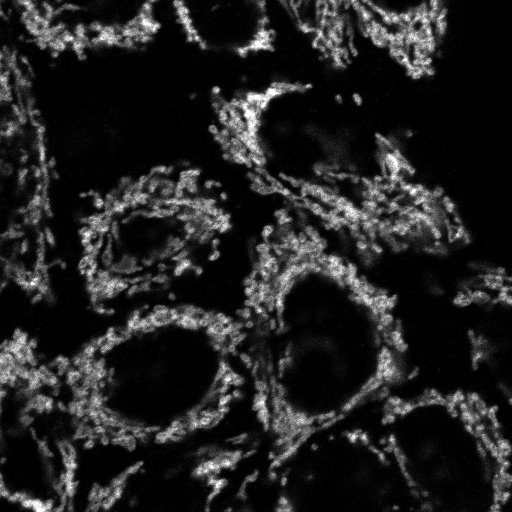

Supplement: Supplementary file 8 — Source Data Fig. 7 [file 44318_2024_36_MOESM8_ESM.zip › Figure7/7A/SourceData_Fig7A_GFP_NT_TOMM20.tif]

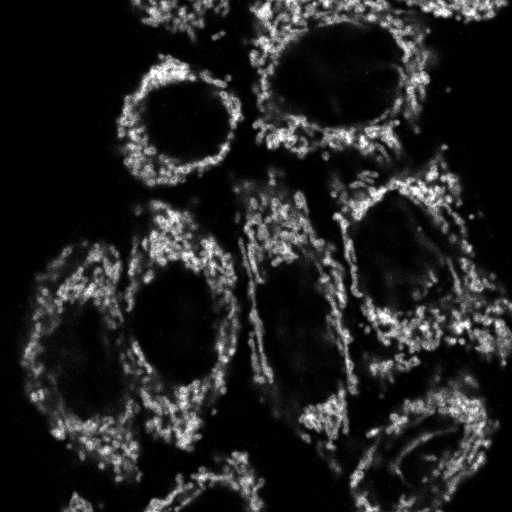

Supplement: Supplementary file 8 — Source Data Fig. 7 [file 44318_2024_36_MOESM8_ESM.zip › Figure7/7A/SourceData_Fig7A_MonoB3_NT_TOMM20.tif]

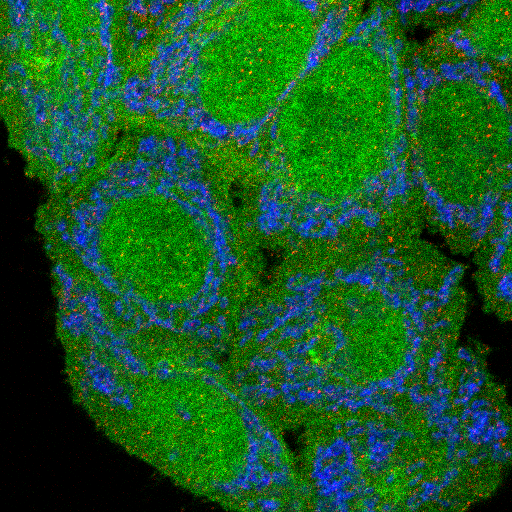

Supplement: Supplementary file 8 — Source Data Fig. 7 [file 44318_2024_36_MOESM8_ESM.zip › Figure7/7A/SourceData_Fig7A_MonoB4_NT_Merge.tif]

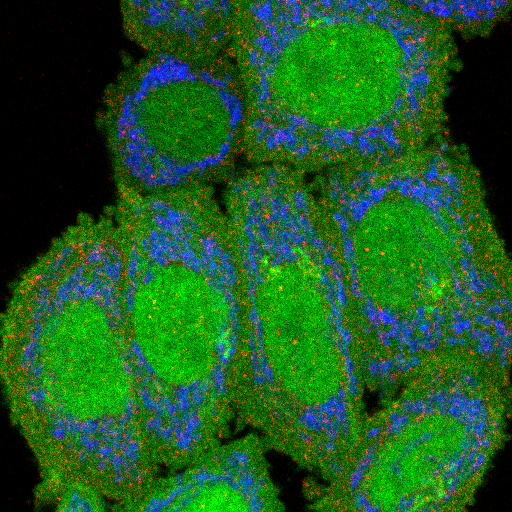

Supplement: Supplementary file 8 — Source Data Fig. 7 [file 44318_2024_36_MOESM8_ESM.zip › Figure7/7A/SourceData_Fig7A_MonoB3_NT_Merge.tif]

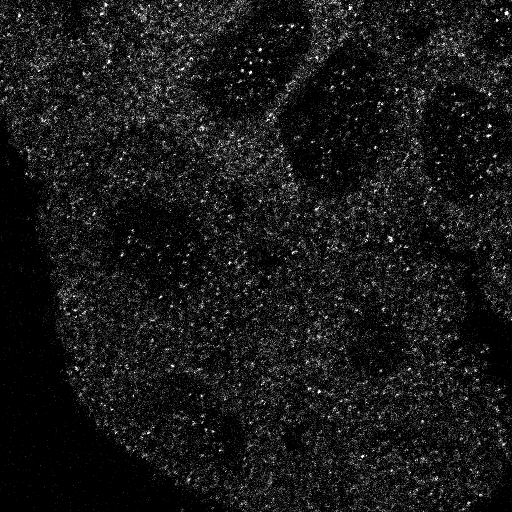

Supplement: Supplementary file 8 — Source Data Fig. 7 [file 44318_2024_36_MOESM8_ESM.zip › Figure7/7A/SourceData_Fig7A_MonoB4_NT_OPTN.tif]

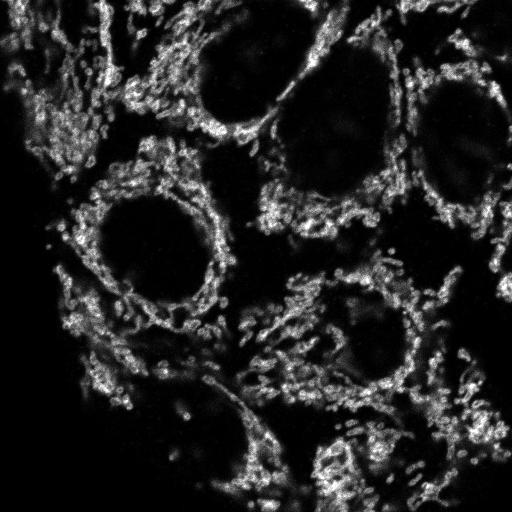

Supplement: Supplementary file 8 — Source Data Fig. 7 [file 44318_2024_36_MOESM8_ESM.zip › Figure7/7A/SourceData_Fig7A_MonoB4_NT_TOMM20.tif]

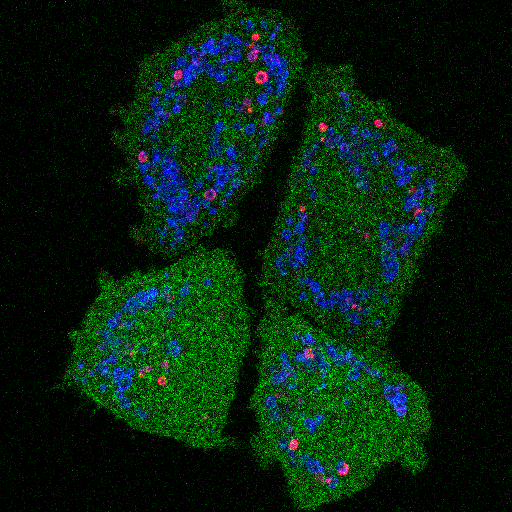

Supplement: Supplementary file 8 — Source Data Fig. 7 [file 44318_2024_36_MOESM8_ESM.zip › Figure7/7A/SourceData_Fig7A_GFP_valbaf90min_Merge.tif]

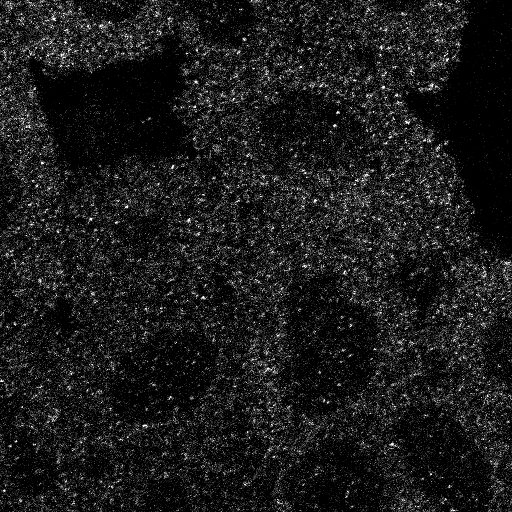

Supplement: Supplementary file 8 — Source Data Fig. 7 [file 44318_2024_36_MOESM8_ESM.zip › Figure7/7A/SourceData_Fig7A_GFP_NT_OPTN.tif]

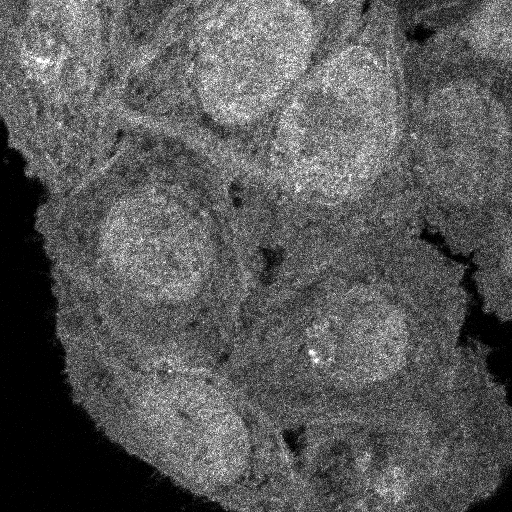

Supplement: Supplementary file 8 — Source Data Fig. 7 [file 44318_2024_36_MOESM8_ESM.zip › Figure7/7A/SourceData_Fig7A_MonoB4_NT_GFP.tif]

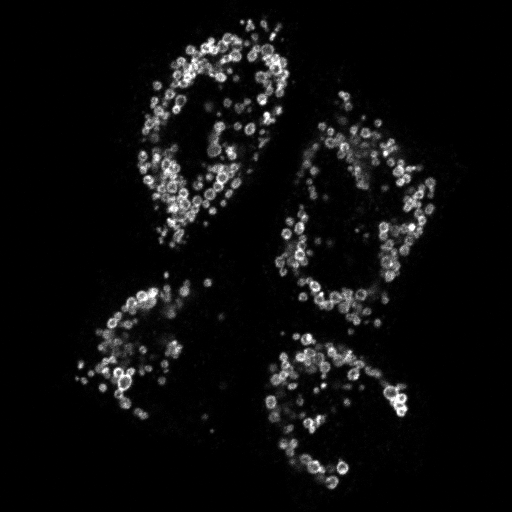

Supplement: Supplementary file 8 — Source Data Fig. 7 [file 44318_2024_36_MOESM8_ESM.zip › Figure7/7A/SourceData_Fig7A_GFP_valbaf90min_TOMM20.tif]

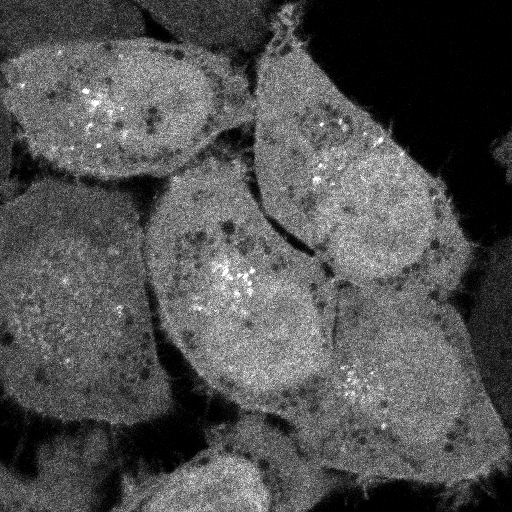

Supplement: Supplementary file 8 — Source Data Fig. 7 [file 44318_2024_36_MOESM8_ESM.zip › Figure7/7A/SourceData_Fig7A_MonoB4_valbaf90min_GFP.tif]

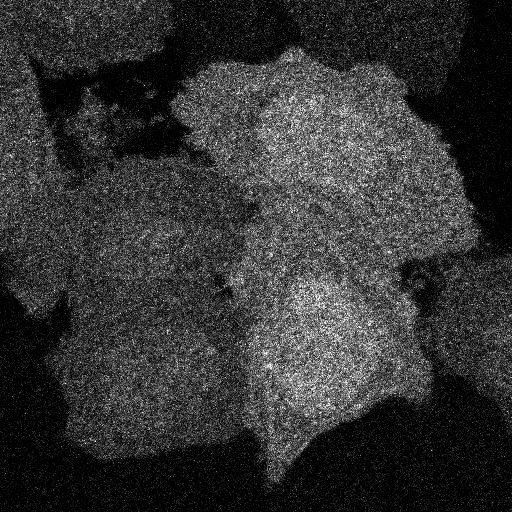

Supplement: Supplementary file 8 — Source Data Fig. 7 [file 44318_2024_36_MOESM8_ESM.zip › Figure7/7A/SourceData_Fig7A_GFP_NT_GFP.tif]

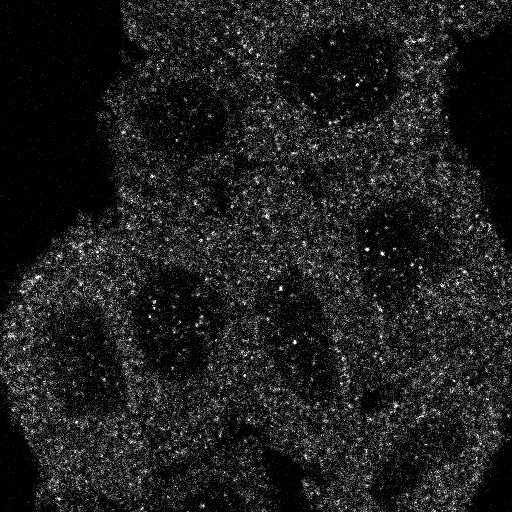

Supplement: Supplementary file 8 — Source Data Fig. 7 [file 44318_2024_36_MOESM8_ESM.zip › Figure7/7A/SourceData_Fig7A_MonoB3_NT_OPTN.tif]

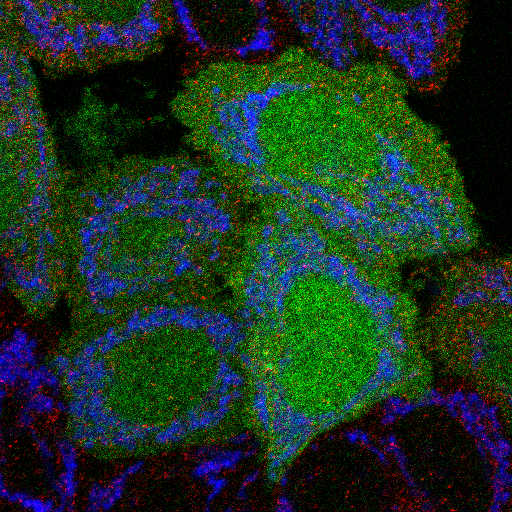

Supplement: Supplementary file 8 — Source Data Fig. 7 [file 44318_2024_36_MOESM8_ESM.zip › Figure7/7A/SourceData_Fig7A_GFP_NT_Merge.tif]

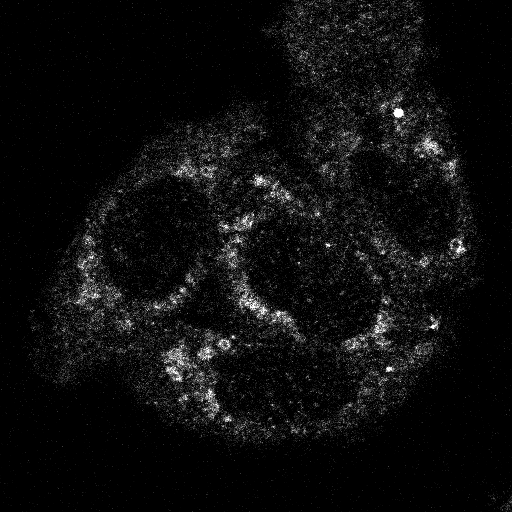

Supplement: Supplementary file 8 — Source Data Fig. 7 [file 44318_2024_36_MOESM8_ESM.zip › Figure7/7A/SourceData_Fig7A_MonoB3_valbaf90min_OPTN.tif]

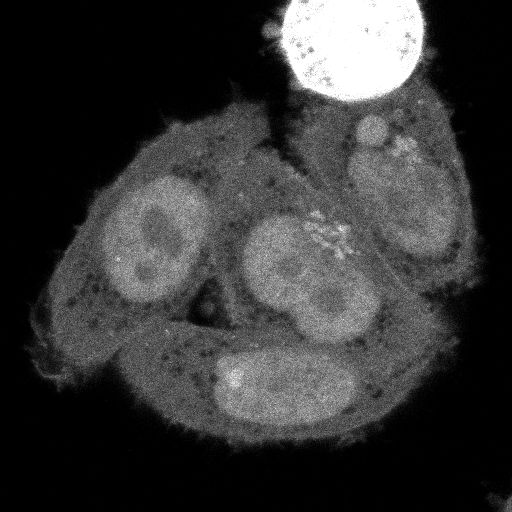

Supplement: Supplementary file 8 — Source Data Fig. 7 [file 44318_2024_36_MOESM8_ESM.zip › Figure7/7A/SourceData_Fig7A_MonoB3_valbaf90min_GFP.tif]

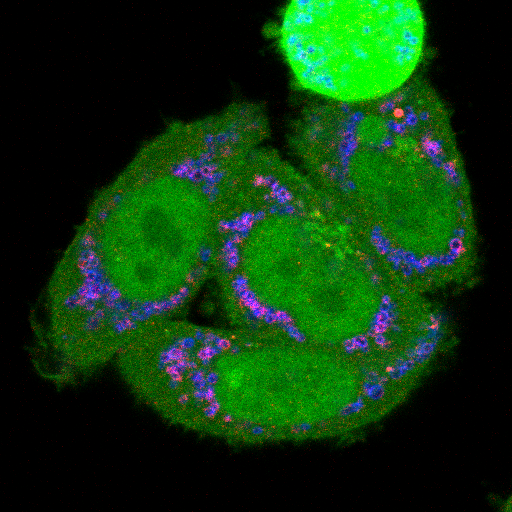

Supplement: Supplementary file 8 — Source Data Fig. 7 [file 44318_2024_36_MOESM8_ESM.zip › Figure7/7A/SourceData_Fig7A_MonoB3_valbaf90min_Merge.tif]
