# Supplementary material for: Optineurin provides a mitophagy contact site for TBK1 activation
Source: EMBO J. 2024 Jan 29;43(5):754–79. doi: 10.1038/s44318-024-00036-1 (PMC10907724; doi:10.1038/s44318-024-00036-1)
Supplement: Supplementary file 12 — Expanded View Figures [file 44318_2024_36_MOESM12_ESM.pdf]

Expanded View Figures

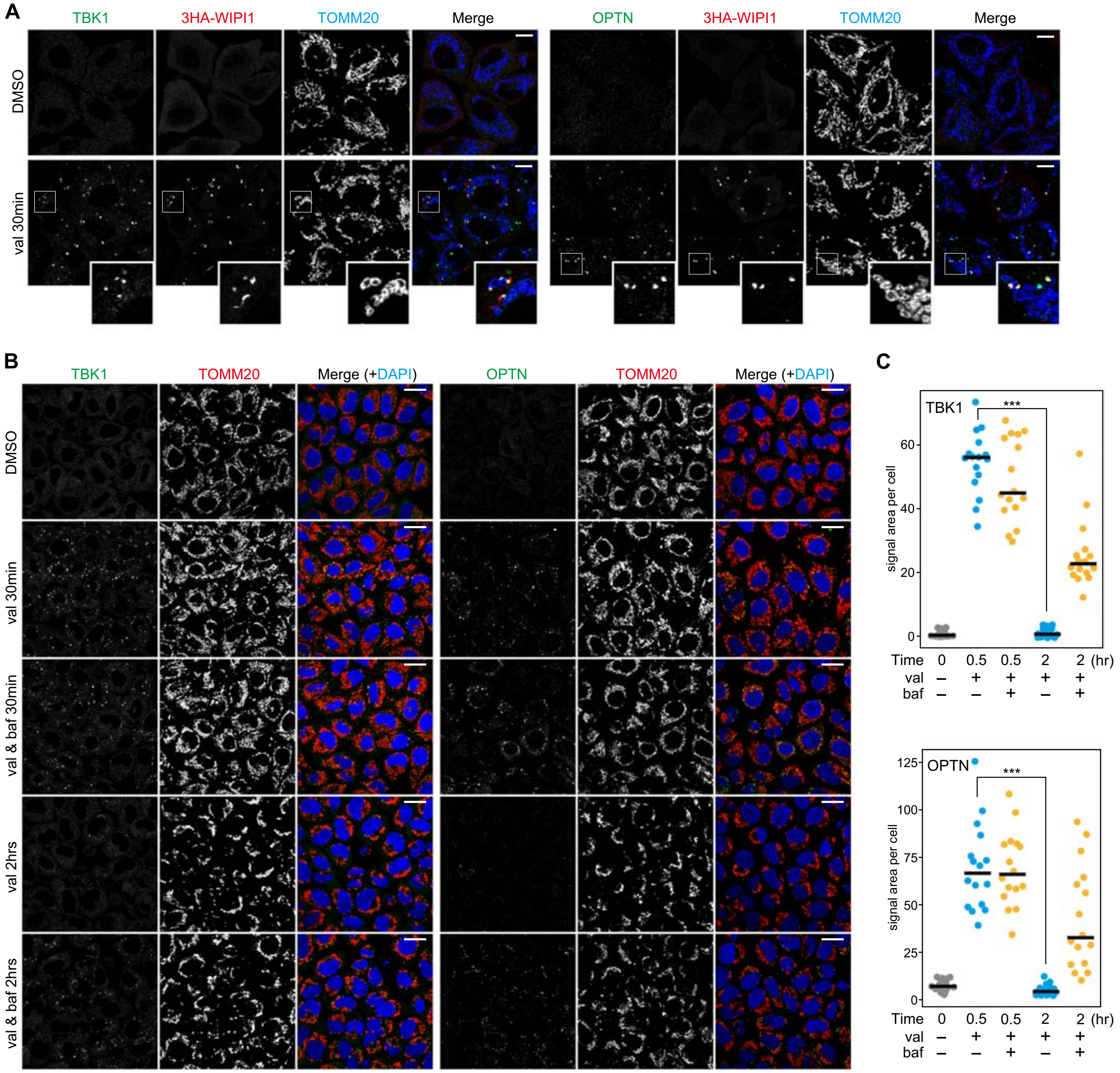

Figure EV1. TBK1 and OPTN recruitments to damaged mitochondria upon Parkin-mediated mitophagy.

(**A**) HeLa cells stably expressing Parkin and 3HA-WIPI1 were treated with DMSO or valinomycin (val) for 30 min and immunostained with the indicated antibodies. Magnified images are also shown for cells treated with val. Bars, 10 µm. (**B**) HeLa cells stably expressing Parkin were treated with DMSO or valinomycin (val) with or without bafilomycin for the indicated times and immunostained with the indicated antibodies. Nuclei were stained with DAPI. Bars, 20 µm. (**C**) The relative areas of TBK1 (upper) and OPTN (lower) foci on mitochondria per cell were quantified. Each dot represents the mean value determined from 18–36 cells, and the horizontal lines indicate the median. ***$P < 0.001$ by two-tailed Student's *t*-test.

© The Author(s)

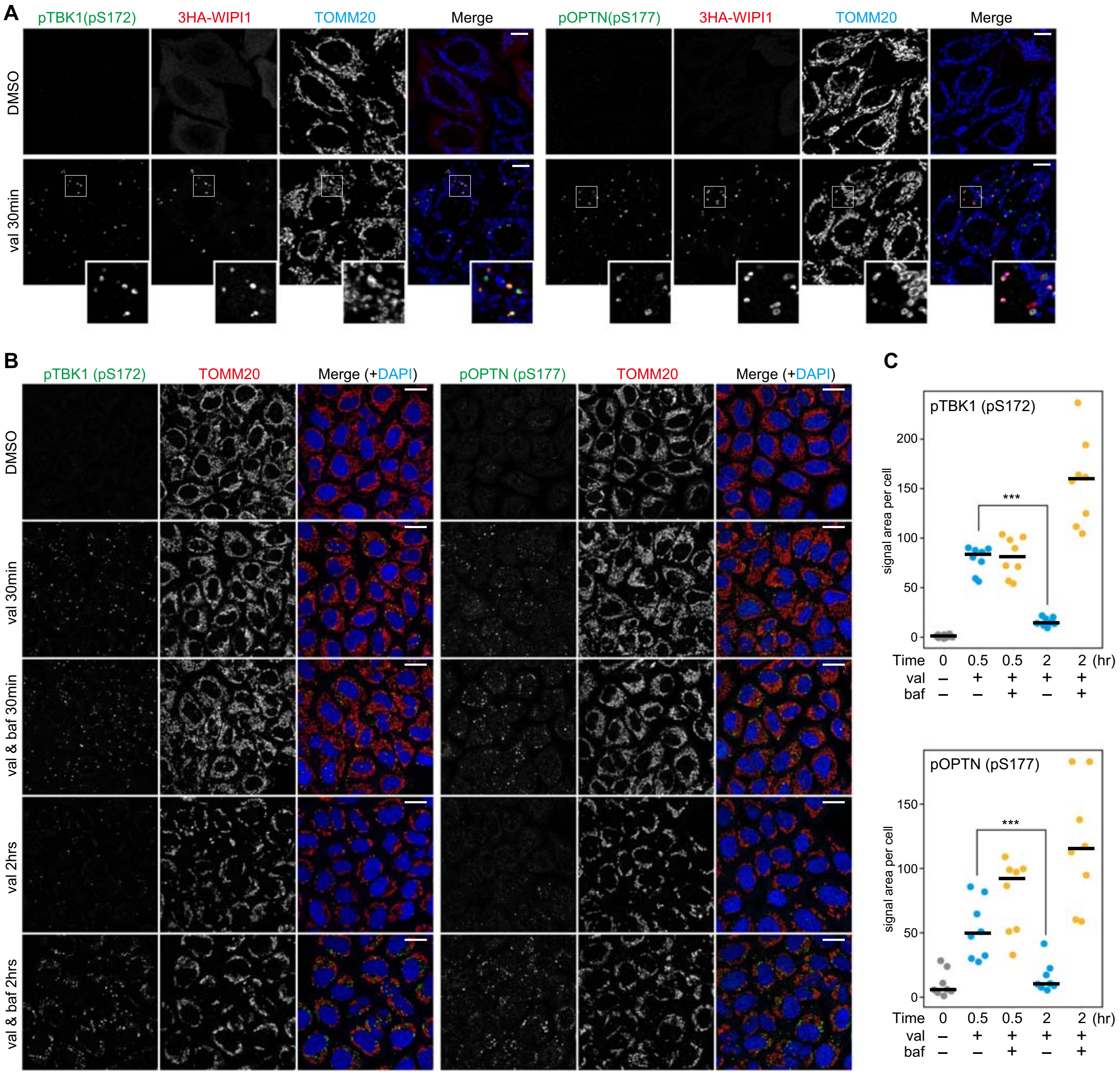

Figure EV2. phosphorylated TBK1 and phosphorylated OPTN productions on damaged mitochondria upon Parkin-mediated mitophagy.

(**A**) HeLa cells stably expressing Parkin and 3HA-WIPI1 were treated with DMSO or valinomycin (val) for 30 min and immunostained with the indicated antibodies. Magnified images are also shown for cells treated with val. Bars, 10 µm. (**B**) HeLa cells stably expressing Parkin were treated with DMSO or valinomycin (val) with or without bafilomycin for the indicated times and immunostained with the indicated antibodies. Nuclei were stained with DAPI. Bars, 20 µm. (**C**) The relative areas of pTBK1(pS172) (upper) and pOPTN(pS177) (lower) foci on mitochondria per cell were quantified. Each dot represents the mean value determined from 18–36 cells, and the horizontal lines indicate the median. ***$P < 0.001$ by two-tailed Student's *t*-test.

 © The Author(s)

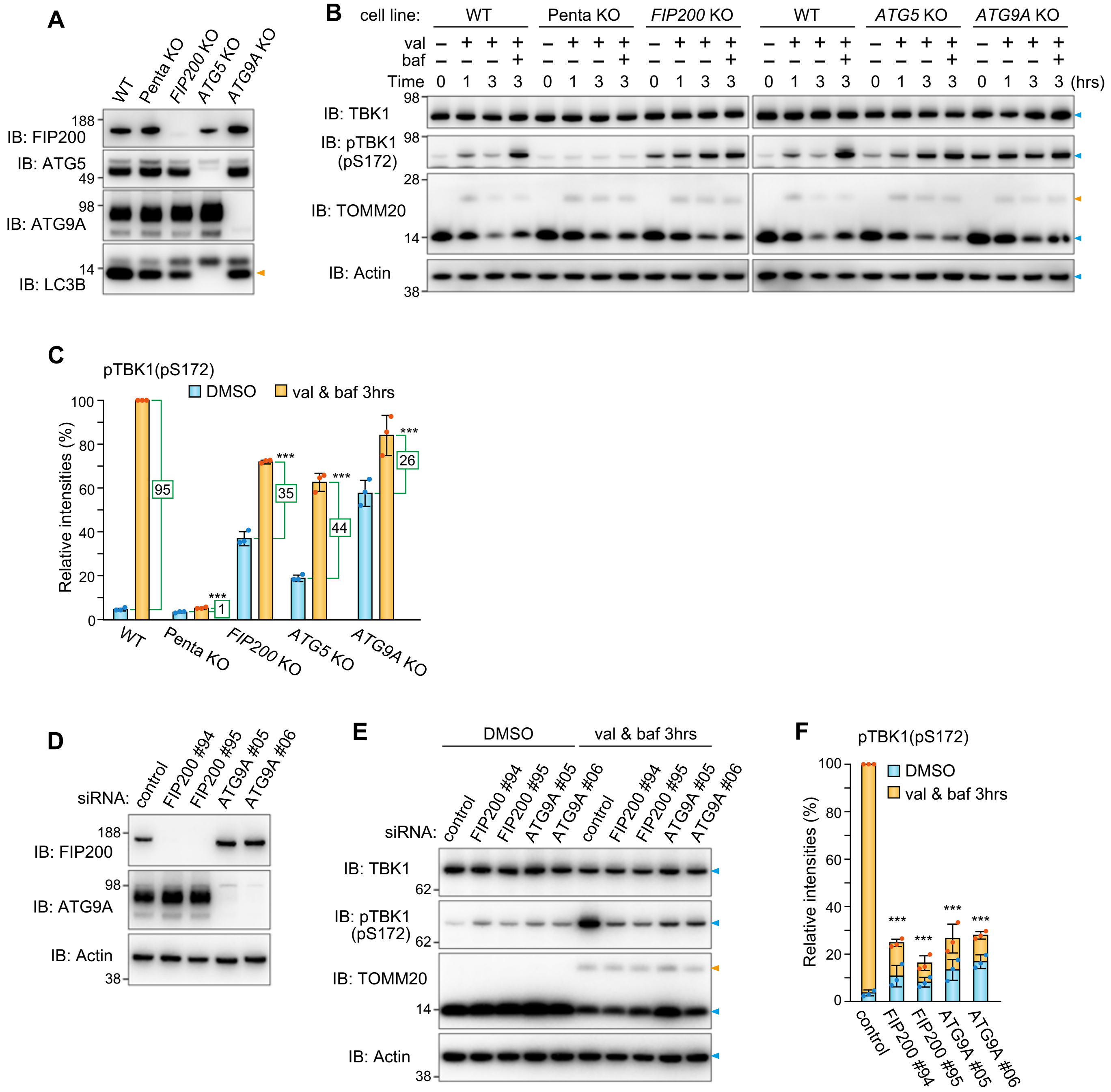

Figure EV3. TBK1 activation during Parkin-mediated mitophagy requires autophagy core components.

(**A**) Depletion of the indicated proteins was confirmed by immunoblotting (IB). The orange arrowhead denotes lipidated forms of LC3B. (**B**) The indicated cells stably expressing Parkin were treated with val and baf for the indicated times. Total cell lysates were analyzed by IB. (**C**) The levels of pTBK1(pS172) in (**B**) were quantified. The level of pTBK1 in WT cells treated with val and baf for 3 h was set to 100. Error bars represent mean ± s.d. of three independent experiments. Signal intensities for pTBK1(pS172) specifically generated during Parkin-mediated mitophagy were determined by subtracting signals for pTBK1 in DMSO from those following val and baf for 3 h (green lines). The difference scores are indicated in the green boxes. (**D**) HeLa cells stably expressing Parkin treated with the indicated siRNA were analyzed by IB. (**E**) HeLa cells stably expressing Parkin pre-treated with the indicated siRNA were treated with val and baf for 3 h and analyzed by IB. (**F**) The levels of pTBK1(pS172) in (**E**) were quantified. The pTBK1 level in control cells was set to 100%. Error bars represent mean ± s.d. of three independent experiments. Data information: ***$P < 0.001$ by two-tailed Dunnett's test (**C**,**F**). The light blue and orange arrowheads indicate unmodified and ubiquitinated bands, respectively (**B**,**E**).

© The Author(s)

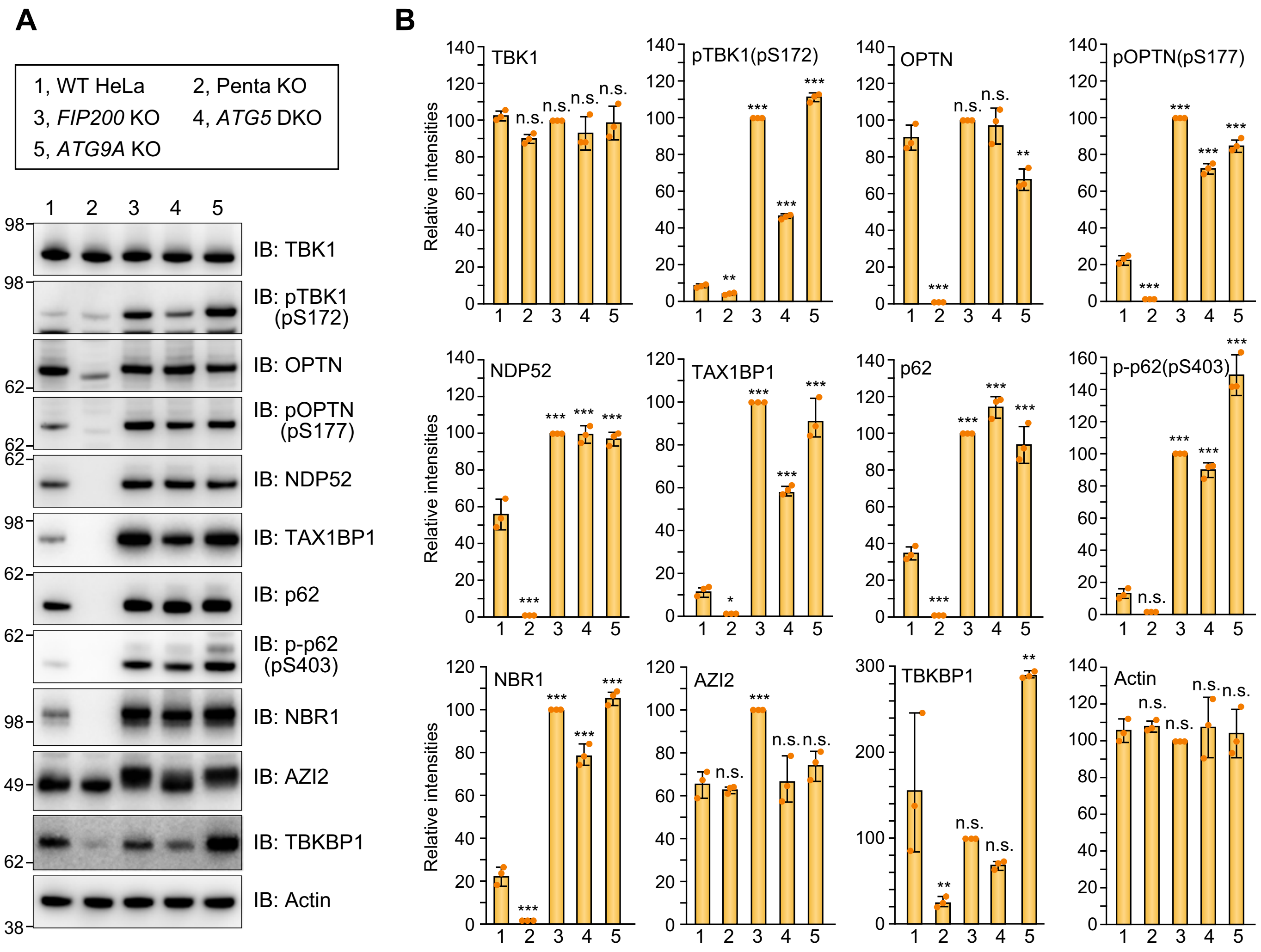

Figure EV4. Accumulations of autophagy adaptors by loss of autophagy core components.

(**A**) The indicated proteins in WT, Penta KO, *FIP200* KO, *ATG5* KO, and *ATG9A* KO HeLa cells were analyzed by immunoblotting. (**B**) Protein levels in (**A**) were quantified. Protein levels in *FIP200* KO cells were set to 100. Error bars represent mean ± s.d. of three independent experiments. n.s. not significant, *$P < 0.05$, **$P < 0.01$, ***$P < 0.001$ by two-tailed Dunnett's test.

 © The Author(s)

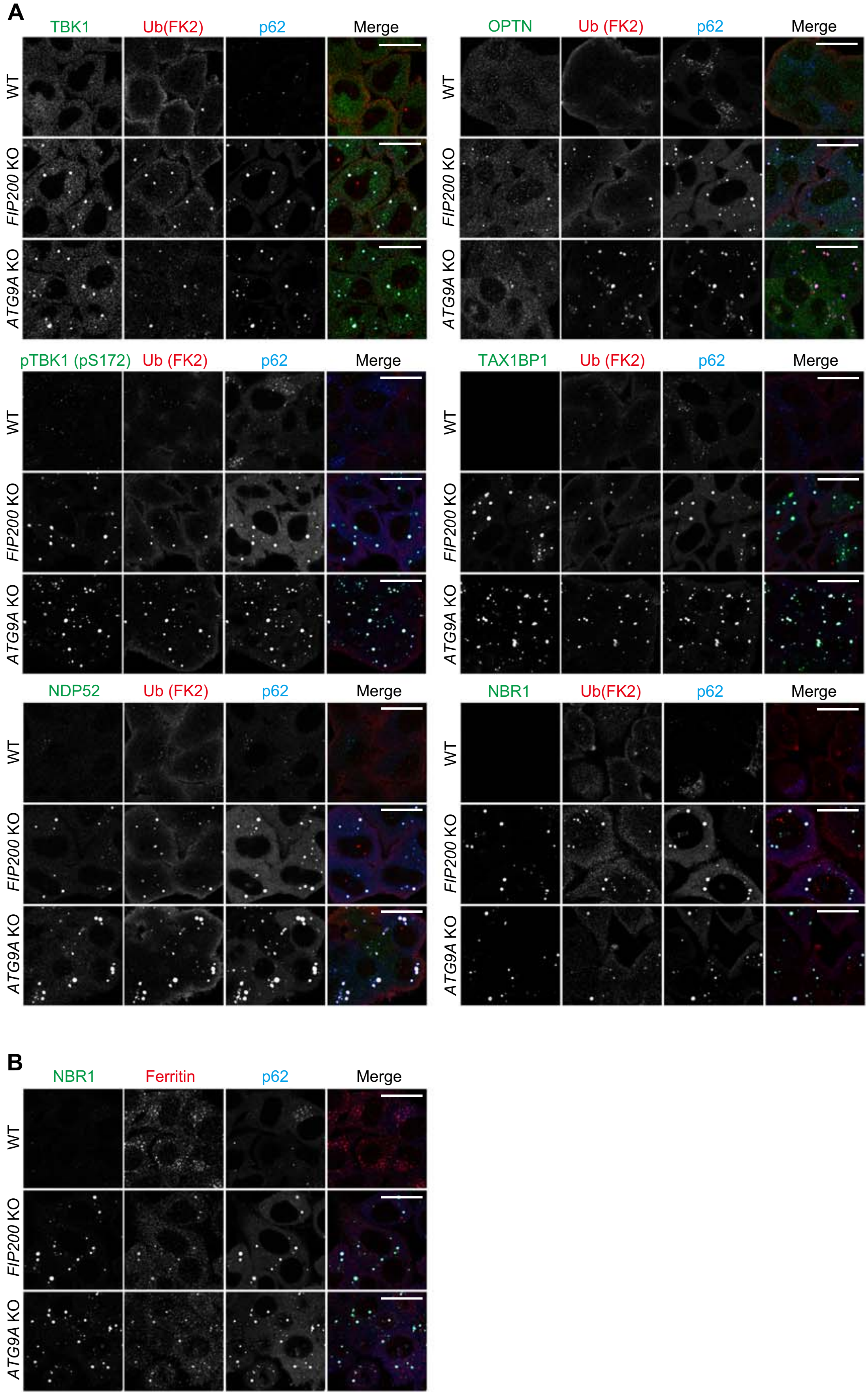
A
TBK1
Ub(FK2)
p62
Merge
WT
FIP200 KO
ATG9A KO
OPTN
Ub (FK2)
p62
Merge
pTBK1 (pS172)
Ub (FK2)
p62
Merge
TAX1BP1
Ub (FK2)
p62
Merge
NDP52
Ub (FK2)
p62
Merge
NBR1
Ub(FK2)
p62
Merge
B
NBR1
Ferritin
p62
Merge

Figure EV5. Accumulations of autophagy adaptors in ubiquitin-positive condensates by loss of autophagy core components.

(**A**,**B**) WT, *FIP200* KO, and *ATG9A* KO HeLa cells were immunostained with the indicated antibodies. Bars, 10 µm.

 © The Author(s)
