# Supplementary material for: Optineurin provides a mitophagy contact site for TBK1 activation
Source: EMBO J. 2024 Jan 29;43(5):754–79. doi: 10.1038/s44318-024-00036-1 (PMC10907724; doi:10.1038/s44318-024-00036-1)

Appendix Fig S1

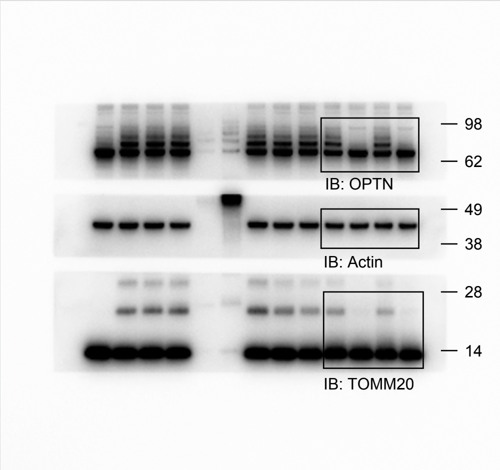

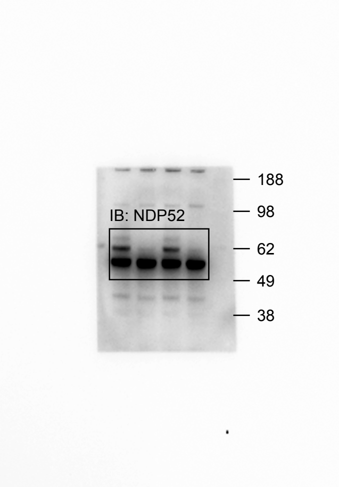

Supplement: Supplementary file 9 — EV and Appendix Figure Source Data [file 44318_2024_36_MOESM9_ESM.zip › SourceData_Appendix_FigureS1.zip!/Appendix FigureS1/SourceData_AppendixFigS1.pdf]
