# Supplementary material for: Optineurin provides a mitophagy contact site for TBK1 activation
Source: EMBO J. 2024 Jan 29;43(5):754–79. doi: 10.1038/s44318-024-00036-1 (PMC10907724; doi:10.1038/s44318-024-00036-1)
Supplement: Supplementary file 9 — EV and Appendix Figure Source Data [file 44318_2024_36_MOESM9_ESM.zip › SourceData_FigureEV3.zip!/FigureEV3/EV3A/SourceData_FigEV3A.pdf]

Fig EV3A

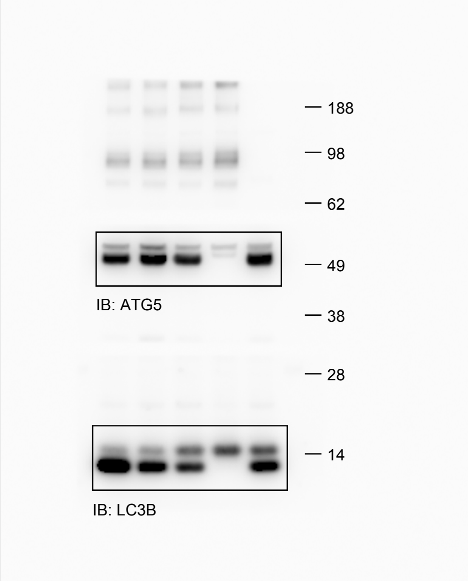
188
98
62
49
IB: ATG5
38
28
14
IB: LC3B

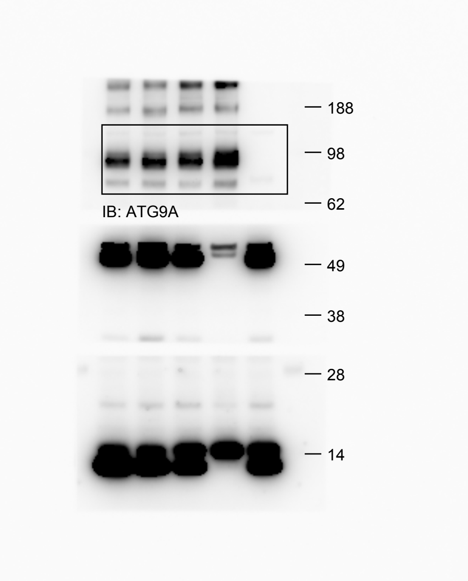
188
98
IB: ATG9A
62
49
38
28
14

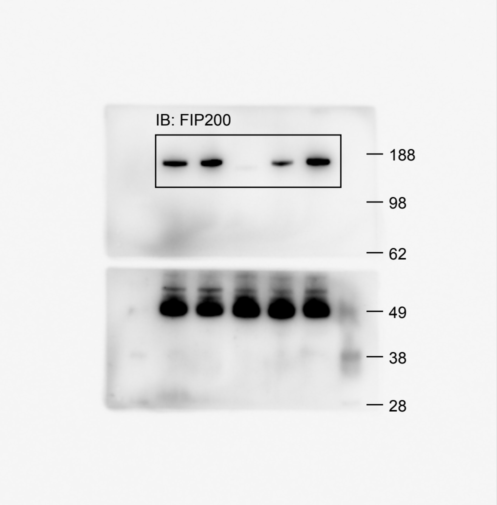
IB: FIP200
188
98
62
49
38
28
