# Supplementary material for: Optineurin provides a mitophagy contact site for TBK1 activation
Source: EMBO J. 2024 Jan 29;43(5):754–79. doi: 10.1038/s44318-024-00036-1 (PMC10907724; doi:10.1038/s44318-024-00036-1)
Supplement: Supplementary file 9 — EV and Appendix Figure Source Data [file 44318_2024_36_MOESM9_ESM.zip › SourceData_FigureEV3.zip!/FigureEV3/EV3B/SourceData_FigEV3B.pdf]

Fig EV3B

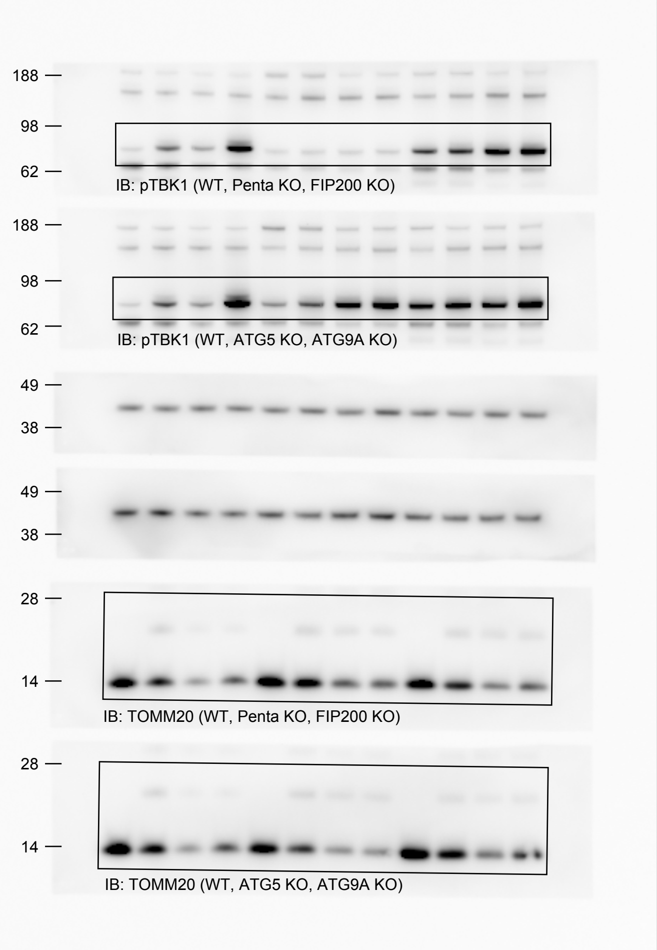
188
98
62
IB: pTBK1 (WT, Penta KO, FIP200 KO)
188
98
62
IB: pTBK1 (WT, ATG5 KO, ATG9A KO)
49
38
49
38
28
14
IB: TOMM20 (WT, Penta KO, FIP200 KO)
28
14
IB: TOMM20 (WT, ATG5 KO, ATG9A KO)

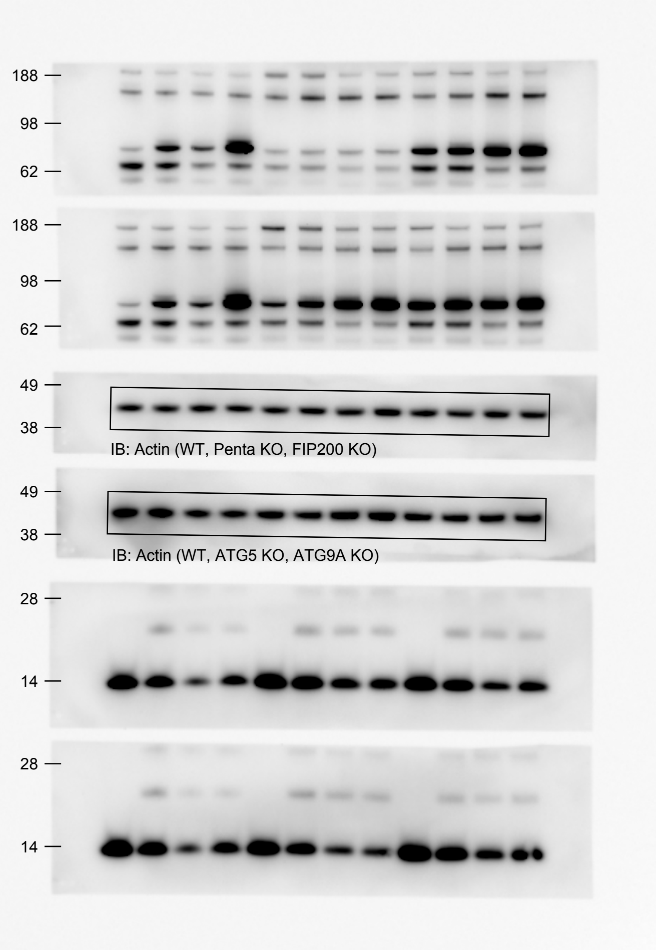
188
98
62
188
98
62
49
38
IB: Actin (WT, Penta KO, FIP200 KO)
49
38
IB: Actin (WT, ATG5 KO, ATG9A KO)
28
14
28
14

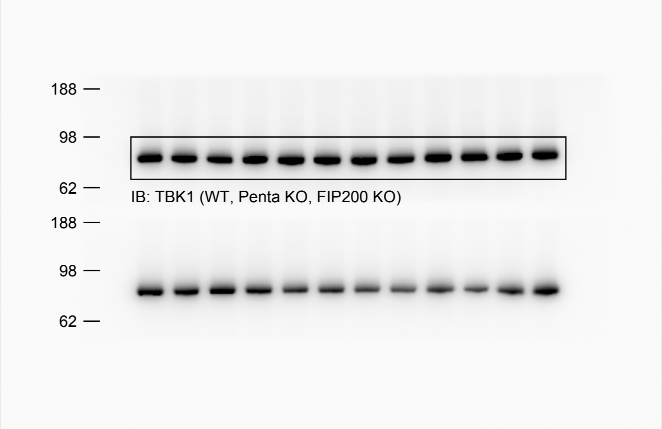
188
98
62
IB: TBK1 (WT, Penta KO, FIP200 KO)
188
98
62

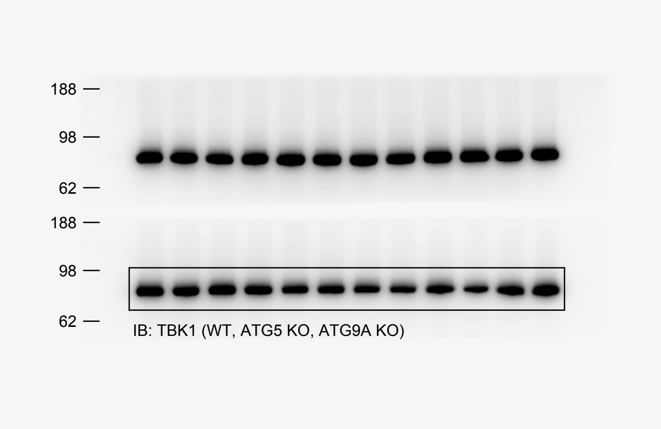
188
98
62
188
98
62
IB: TBK1 (WT, ATG5 KO, ATG9A KO)
