# Supplementary material for: Optineurin provides a mitophagy contact site for TBK1 activation
Source: EMBO J. 2024 Jan 29;43(5):754–79. doi: 10.1038/s44318-024-00036-1 (PMC10907724; doi:10.1038/s44318-024-00036-1)
Supplement: Supplementary file 9 — EV and Appendix Figure Source Data [file 44318_2024_36_MOESM9_ESM.zip › SourceData_FigureEV3.zip!/FigureEV3/EV3E/SourceData_FigEV3E.pdf]

Fig EV3E

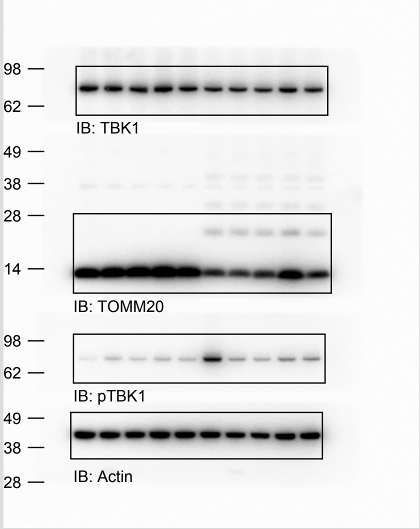
98
62
IB: TBK1
49
38
28
14
IB: TOMM20
98
62
IB: pTBK1
49
38
28
IB: Actin
