# Supplementary material for: Optineurin provides a mitophagy contact site for TBK1 activation
Source: EMBO J. 2024 Jan 29;43(5):754–79. doi: 10.1038/s44318-024-00036-1 (PMC10907724; doi:10.1038/s44318-024-00036-1)
Supplement: Supplementary file 9 — EV and Appendix Figure Source Data [file 44318_2024_36_MOESM9_ESM.zip › SourceData_FigureEV4.zip!/FigureEV4/EV4A/SourceData_FigEV4A.pdf]

Fig EV4A

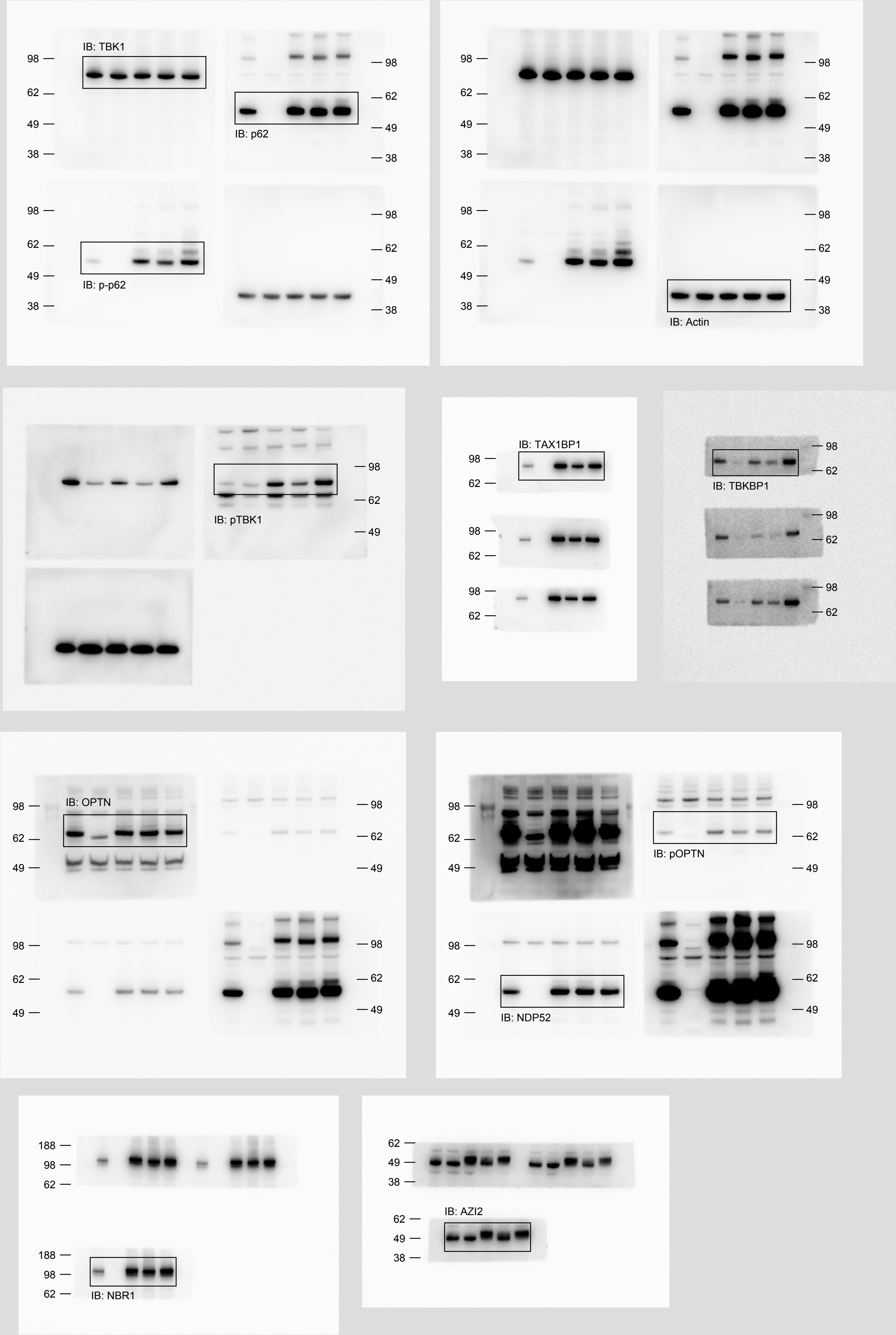
IB: TBK1
IB: p62
IB: p-p62
IB: Actin
IB: pTBK1
IB: TAX1BP1
IB: TBKBP1
IB: OPTN
IB: pOPTN
IB: NDP52
IB: NBR1
IB: AZI2
188
98
62
49
38
